# Multifunctional cobalt ferrite nanoparticles with optimized cobalt doping for enhanced photo-Fenton catalysis, energy storage, and antifungal applications

**DOI:** 10.1038/s41598-026-55577-8

**Published:** 2026-06-14

**Authors:** Rupali Chavan, Shruti Deshpande, Jayram Shelke, Vijay Chavan, Mahendra Waghmare, Prashant Patil, Jyoti Jadhav, Rahul Patil, Deok-kee Kim, Ashok Chougale

**Affiliations:** 1 Department of Chemistry, The New College Kolhapur, Shivaji University, Kolhapur, India; 2 Department of Physics, The New College Kolhapur, Shivaji University, Kolhapur, India; 3 Department of Botany, The New College Kolhapur, Shivaji University, Kolhapur, India; 4 Department of Semiconductor Systems Engineering, Sejong University, Seoul, Republic of Korea; 5 Department of Biochemistry, Shivaji University, Kolhapur, India; 6 Department of Physics, Yashwantrao Patil Science College, Shivaji University, Solankur, Kolhapur, India

**Keywords:** Cobalt ferrites, Dye degradation, Magnetic nanoparticles, Energy storage, Antifungal activity, Chemistry, Engineering, Environmental sciences, Materials science, Nanoscience and technology

## Abstract

Designing cost-effective, eco-friendly, and high-performance nanomaterials remains crucial for advancing next-generation technologies in environmental remediation, energy conversion, and pathogen control. Here, we report the glucose-assisted synthesis of cobalt ferrite (CoFe) nanocomposites (NCs) with tunable Co concentrations (0.025–0.15 M), and utilized for multifunctional applications. XRD confirmed the phase-pure cubic spinel ferrite, and FESEM showed a morphological transition from irregular aggregates to well-defined hexagonal shapes, at the optimal dopant concentration. Among the four batches, CoFe-3 (0.1 M Co) exhibited the highest surface area (74.98 m²/g) with the pore size (16.17 nm), contributing to versatility. CoFe-3 achieved 91.44% efficiency for crystal violet (CV) degradation within 40 min under photo-Fenton conditions and retained 81.73% efficiency after five cycles. Electrochemical studies displayed a high specific capacitance of 614 F/g at 1 A/g with stable cycling over 3000 GCD cycles, indicating battery-type behaviour with promising energy (15.04 Wh/kg) and power densities (217.16 W/kg). Moreover, CoFe-3 displayed significant antifungal activity against *Fusarium solani*, inducing morphological deformities in hyphae. These results highlight the potential of glucose-engineered CoFe NCs as robust materials for environmental remediation, energy storage, and antifungal applications.

**Supplementary Information:**

The online version contains supplementary material available at https://doi.org/10.1038/s41598-026-55577-8.

## Introduction

The global demands for clean water, energy, and effective pathogen control technologies are getting accelerated. However, continuous release of industrial pollutant causes significant environmental deterioration. The widespread release of synthetic dyes generates persistent organic contaminations that resist conventional degradation. Owing their toxicity, carcinogenicity, and long-term bioaccumulation, cationic dyes like methylene blue, crystal violet (CV), etc. lead to serious health risks to marine and terrestrial organisms, even at minimal trace amounts^1^. Conventional wastewater treatment methods, including anaerobic digestion as well as physical and biological methods, such as adsorption, ion exchange, flocculation, and microbiological treatment using bacteria, fungi, and yeast, have been employed to treat these industrial pollutants^2,3^. Despite their utility, they suffer from slow kinetics, strict operational conditions, and limited efficiency towards the recalcitrant pollutant. Additionally, physical methods generate non-disposable solid waste, whereas biological methods have limited applicability due to their stringent conditions^4–6^. Thus, in the current era, chemical methods, advanced oxidation methods (AOPs) are mostly preferred to overcome drawbacks of non-disposable solid waste^7–9^. Among various AOPs, photocatalysis^10^, Fenton and photo-Fenton are widely recognized for their excellent performance in wastewater treatment. Light driven photocatalysis induces reactive oxygen species (ROS)^11,12^. In contrast, the conventional Fenton system produces ^●^OH radical via Fe^2+^ and H_2_O_2_^3^, while the photo-Fenton process boosts this activity under light irradiation which further amplifies the catalytic activity^13^. Other reducing agents such as NABH_4_^14^, peroxymonosulfate^15^ can also participate in the catalytic activation and pollutant removal; however, H_2_O_2_ is still the most extensively used oxidant due to its high efficiency, operational simplicity, and compatibility with metal oxide catalysts^13^.

Parallel to the environmental challenges, sustainable global expansion has raised the advancement and need for high-performance energy-storage materials and devices to provide energy-related mandates^16^. In particular, supercapacitors (SCs) have gained prominence due to their notable advantages, including high power, cyclic stability, and environmental safety^17^. The exceptionally high-power density and reversibility of SCs enable their broad use across energy and electronic technologies. However, the physicochemical properties of the electrode material have significantly influenced the electrochemical performance, which requires a nanomaterial with optimal surface chemistry, conductivity, and redox activity. Recent studies show that the structural and morphological modulation of mixed oxide nanostructures, such as porous architectures and composite engineering, can significantly enhance conductivity, ion diffusion pathway, redox activity, and cycling stability in advanced energy-storage systems^18–22^. These findings highlight the importance of structural modification in developing multifunctional electrode material with enhanced charge-storage capacity.

Heightened antimicrobial resistance and the growing incidence of fungal infections further strain the environment and energy infrastructure, as pathogenic fungi like *Fusarium oxysporum*,* Neofusicoccum* spp., and *Fusarium solani* create a widespread threat to agricultural and forest species, resulting in economic loss and ecological disturbance. Addressing this issue of environmental pollution, energy crisis and pathogenic infection is therefore critical, as conventional fungicides suffer from persistence, low selectivity, and resistance, prompting the growing interest of nanomaterials with intrinsic or stimulus-responsive antifungal activity.

The functionality of the SCs, photo-Fenton reaction, and antibacterial activity largely hinges on the catalyst, with its inherent properties primarily governed its effectiveness^23^. Earlier, numerous catalysts were tested against applications such as photo-Fenton reaction, SCs, and antibacterial activity, respectively. Lu et al. demonstrated catalytic degradation of p-nitrophenol using Sb_2_O_3_-CuO NCs^24^. NiO and Ag@NiO nanoparticles (NPs) determining their SCs performance and degradation of fast blue (FB) dye^25^. The synergistic effects on photocatalytic and electrochemical energy storage applications, as well as antibacterial activity were investigated by porous CuO NPs^26^. Due to the post-treatment recovery issues with non-magnetic NPs, magnetic metal oxide NPs have garned considerable attention from researchers. Somasundaram et al. demonstrated MnFe_2_O_4_/Polyaniline nanocomposites for SCs and photocatalytic applications^27^. Chavan et al. synthesized Zr^2+^ doped CuFe_2_O_4_ with a stable spinal structure showing enhanced antibacterial properties^28^. Kharat et al. exhibited heightened supercapacitor performance of the nickel-zinc ferrite owing to their multiple redox sites and favourable conductivity^29^. El‑Khawaga et al. conveyed considerable antibacterial activity and UV-assisted photocatalytic activity of MgFe_2_O_4_^30^. Among the other magnetic metal oxide NCs, cobalt ferrites (CoFe) are the most substantial group^31^. CoFe illustrates an efficient synthesis method, owing to its chemical durability, extensive anisotropy constant, an array of oxidoreduction states, extra operative electrochemical strength, and a highly magnetostrictive nature^31^. CoFe exhibits narrow bandgap which enables the visible light absorption, while mixed Co and Fe valence states promotes the redox activity (Fe^2+^/Fe^3+^, Co^2+^/Co^3+^)^32^ and ROS formation^33^. Such an exclusive physical, chemical, and electrochemical properties of CoFe is used as a model material for executing versatile applications.

Although significant research has been conducted on cobalt ferrites, there is still a lack of a clear understanding of how compositional tuning can control multifunctional operation in environmental, electrochemical, and biological applications. This research paper fills this gap by conducting systematic research to explore the role of cobalt concentration in the modulation of structural, electronic and surface properties of CoFe nanocomposites. The main research questions are: (i) how the compositional variation affects phase evolution and defect structure, (ii) how these variations affect charge transfer and reactive species generation, and (iii) how a single optimized composition can be used to provide better multifunctional performance. Previously, investigations on CoFe have been largely focused on the single functional aspect, leaving their boarder multifunctional performance insufficiently examined across the environmental, electrochemical and biomedical aspects. Thus, comprehensive evaluation of the CoFe encompassing dye degradation, SCs and antifungal activity were systematically investigated in this study. This research explores the relationship between structural features and defect sites and their influence on the multifunctional catalytic system. It is hypothesized that compositional tuning and defect engineering in CoFe will promote charge carrier separation and reactive species generation, thereby enhancing its activity. Especially antifungal potential of the CoFe was scarcely documented, which have been reported for the first time in this study; underscoring broadening prospect of their applications. Additionally, the current work aligns with the Sustainable Development Goals (SDGs) of the United Nations, including SDG 7(affordable and clean energy) and SDG 13 (climate action). The proposed CoFe-based electrode material with its simple and scalable synthesis technique, has the potential for long-term and cost-effective energy storage application with minimal environmental impact. The series of CoFe batches (CoFe-1 to CoFe-4) were synthesized by the co-precipitation method. The green synthesized CoFe NCs were characterised using XRD, FESEM, TEM, EDS, and BET. Furthermore, the CoFe-3 sample was evaluated for its multifunctional performance, including dye degradation, energy-storage behaviour, and antifungal activity.

### Experimental design

The details regarding the Materials and experimental procedures are described in supporting information.

## Results and discussion

**Fig. 1:**
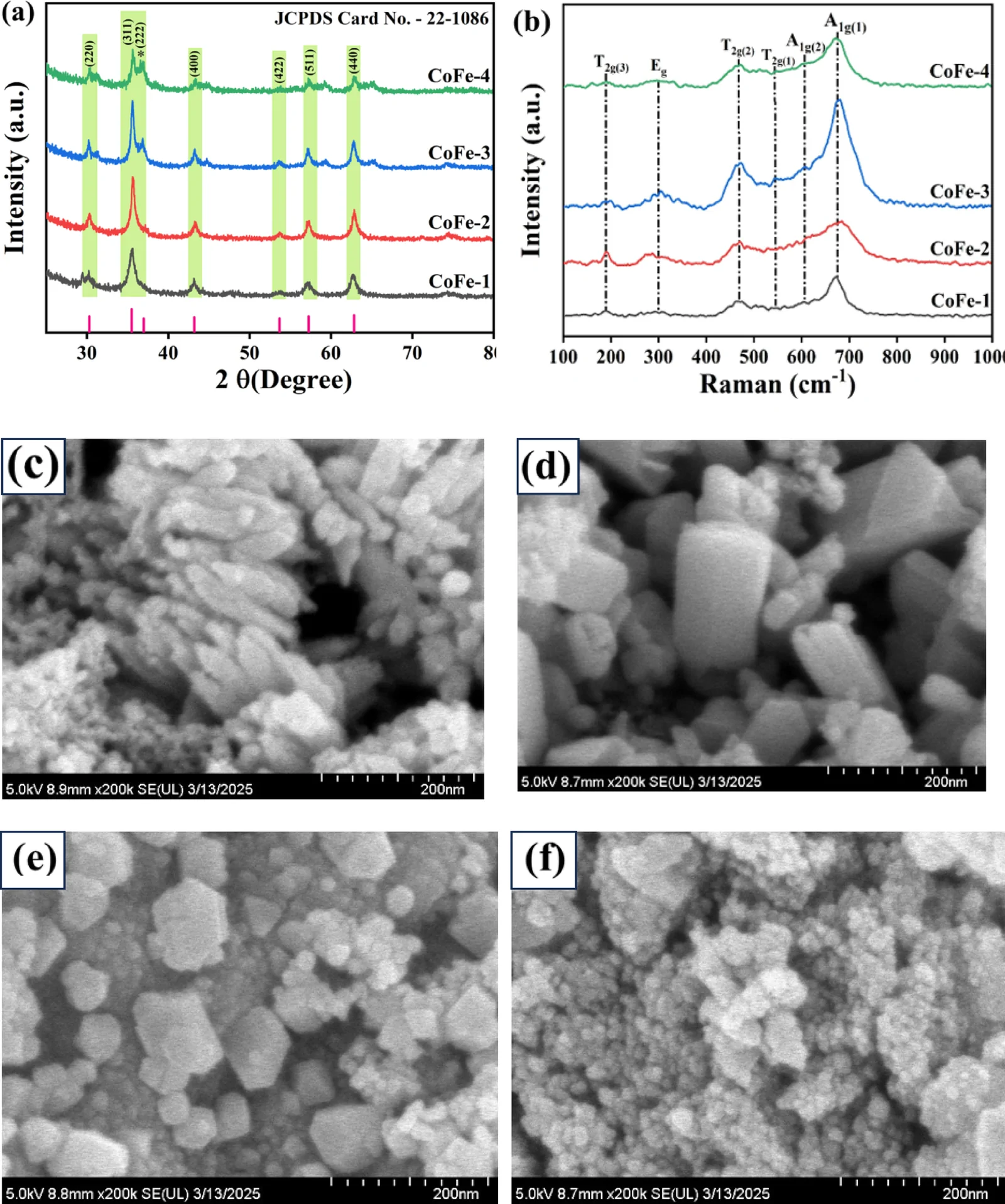
(**a**) XRD diffractogram; (**b**) Raman spectra indicating vibrational modes of CoFe-1, CoFe-2, CoFe-3, and CoFe-4; FESEM images of series of CoFe batches (**c**) CoFe-1, (**d**) CoFe-2, (**e**) CoFe-3, and (**f**) CoFe-4, respectively.

The XRD analysis demonstrated the phases purity and phase evolution in CoFe samples as a function of precursor concentration. Fig. 1a illustrates the XRD spectra of CoFe-1, CoFe-2, CoFe-3, and CoFe-4, respectively. The (hkl) planes (220), (311), (222), (400), (422), (511), and (440) proves a cubic structure of CoFe_2_O_4_. Interestingly, the effect of Co concentration is clearly verified within 2 concentration ranges. At lower Co incorporation in CoFe-1 and CoFe-2 (0.025 M and 0.05 M), the sample revealed the diffraction peaks corresponds to the Fe_3_O_4_. In this scenario, the Co ions can be successfully incorporated in the matrix of Fe_3_O_4_^34^. As further increase in the Co concentration from 0.1 M to 0.15 M (CoFe-3 and CoFe-4), the phase transition from Fe_3_O_4_ to CoFe_2_O_4_ occurred successfully. The relatively intense peaks represented by the CoFe-3 and CoFe-4 batches resemble those on the JCPDS card number (22–1086)^35^. This confirms the CoFe possesses cubic structure without any additional impurities. The optimization of phase and nanostructure development is a key strategy for tailoring structure-property relationship and enhancing functional performance as reported by Yu et al^36^. Likewise, the compositional changes affect the crystallinity and electronic structure, thereby improving the catalytic and electrochemical activity.

The crystallite size dimension of CoFe-1, CoFe-2, CoFe-3, and CoFe-4 were obtained by employing both Scherrer (Eq. 1)^37^ and Williamson-Hall (W-H) methods (Eq. 2)^38^. Using the Scherrer approach, the crystallite sizes found to be 8.77 nm, 13.53 nm, 15.21 nm, and 4 nm, whereas W-H analysis yielded 1.55 nm, 4.82 nm, 36.18 nm, and 17.76 nm. The observed difference between the two methods occurred due to the distinct theoretical principals of the two methods. In the Scherrer model, the peak broadening caused by crystallite size, while W-H method includes the combine effect of size and lattice strain^38^. Among all samples, CoFe-3 exhibits a considerable increase in the crystallite size obtained from W-H method (36.18 nm) compared to the Scherrer value (15.21 nm), indicating size induced broadening with relatively low lattice strain and improved crystal quality. Conversely, the lower W-H values observed for CoFe-1 and CoFe-2 suggest a significance contribution of lattice strain and structural defects. The improved crystallinity and lower lattice strain on CoFe-3 may support efficient charge transport, which may result in enhanced catalytic and electrochemical performance. Additionally, the lattice constant (Eq. 3) of CoFe-1, CoFe-2, CoFe-3, and CoFe-4 were estimated to be 29.08, 44.87, 50.44, and 13.26 Å, respectively^39,40^. From this values, the metal-oxygen bond distances at tetrahedral (A) (Eq. 4) and octahedral (B) sites (Eq. 5) were determined^41^. The calculated tetrahedral bond lengths were found to be 6.28, 9.70, 10.90, and 2.86 Å, while the corresponding octahedral bond lengths were 14.54, 22.43, 25.22, and 6.63 Å, respectively. Particularly, CoFe-3 larger lattice constant and bond lengths, suggesting lattice expansion and altered metal-oxygen interactions that may boost charge transfer and catalytic performance^42^.

1 $$\:D = \frac{{K\lambda \:}}{{\beta COS\:\theta \:}}$$

2 $${\beta _r} = {\beta _D} + {\beta _\tau }$$

3 $$\:a=d\:\sqrt{{h}^{2}+\:{k}^{2}+\:{l}^{2}}$$

4 $$\:{R}_{A}=a\sqrt{3}\:(u-0.25)$$

5 $$\:{R}_{B}=a\sqrt{(0.625-u)}$$

These results show the phase evolution with composition. The replacement of the Fe_3_O_4_dominant phases by the pure CoFe_2_O_4_ at the increased Co concentration (CoFe-3) proves that the crystallinity and the number of defects is the controlled aspects of doping, which are essential to the improvement of charge transport and catalytic properties.

**Fig. 2:**
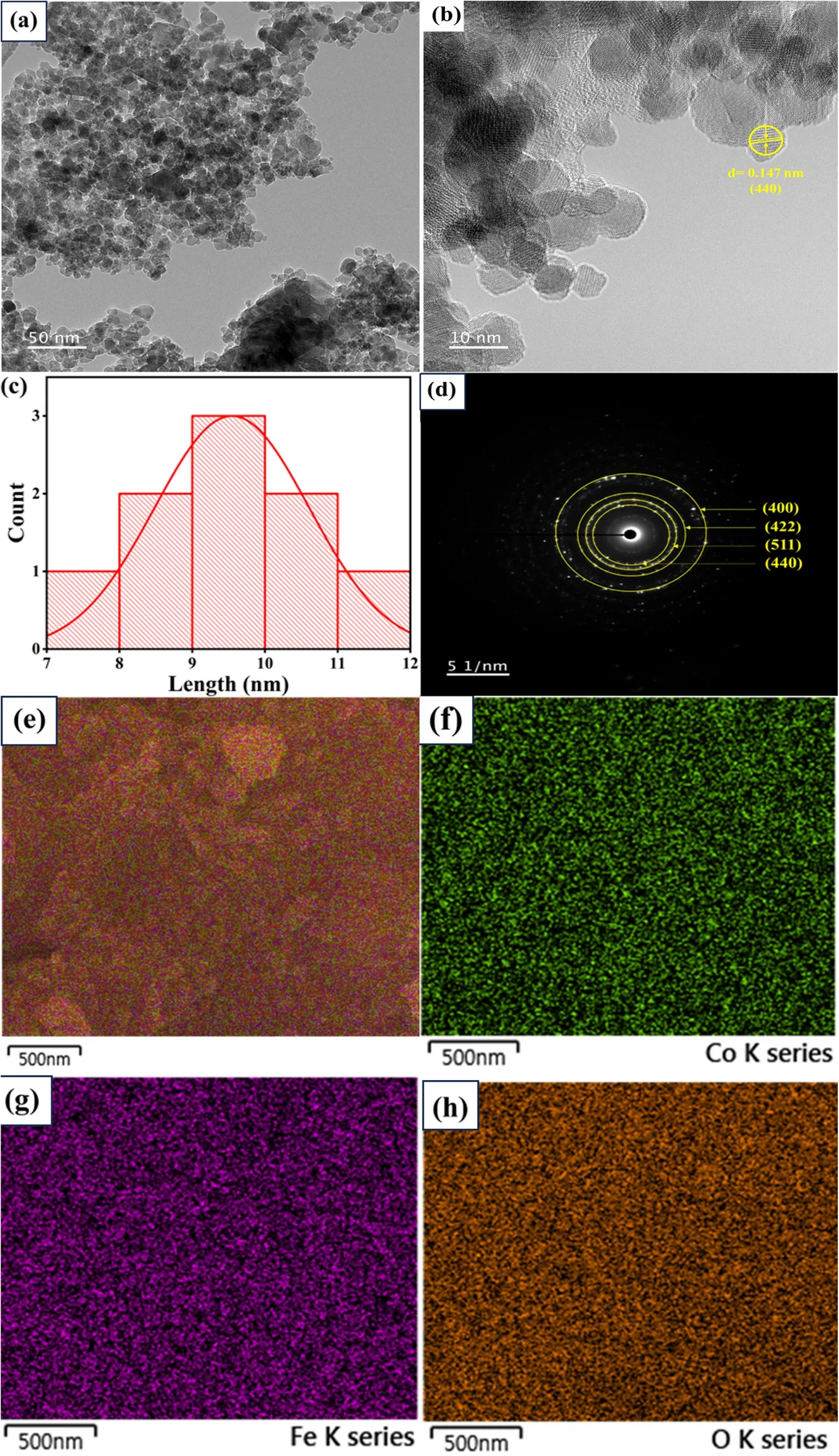
(**a**) TEM image, (**b**) HRTEM image, (**c**) Particle size distribution curve, (**d**) SEAD pattern; Elemental mapping of CoFe-3 (**e**) CoFe, (**f**) Co, (**g**) Fe, and (**h**) O, respectively.

Raman spectroscopy serves as an efficient analytical tool for examining the atomic-scale structure of nanoparticles. Raman spectra corresponding to CoFe-1, CoFe-2, CoFe-3, and CoFe-4 NCs are shown in Fig. 1b. According to group theory, the optical phonon modes are expressed as 5T_1u_ + A_1g_ + E_g_ + 3T_2g_. Among these, 5T_1u_ are active in the infrared region, whereas the other modes are (A_1g_ + E_g_ + 3T_2g_) Raman active and associated with the vibrational movement of oxygen and A- and B- site cations in the spinal framework^43^. Moreover, the symmetric stretching and bending of the oxygen anions are represented by A_1g_ and E_g_ vibrations, while their asymmetric stretching related to the tetrahedral and octahedral cations is associated with the T_2g_ mode^44^. As illustrated in Fig. 2b, the as-synthesized NCs display the Raman modes at ~ 184, ~303, ~ 470, ~544, ~ 607, and ~ 679 cm^− 1^. A shoulder-like feature at lower wavenumber ~ 607 cm^− 1^ alongside the Raman mode ~ 679 cm^− 1^ evidences the A_1g_(1) and A_1g_(2) vibrational modes, indicating the stretching vibration Fe-O and M-O bonds within the tetrahedral sites^45^. The Raman peaks are located at the lower frequency region at ~ 184, ~303, ~ 470, and ~ 544 cm^− 1^, corresponding to the E_g_ and T_2g_ vibrations, reflecting the vibrational characteristics of the spinal structure^45^.

The dye degradation capability depends on the catalyst morphology, which was analyzed via FESEM of the prepared CoFe samples. FESEM micrographs captured at 200 nm (Fig. 1c-f) reveals that the variations in the Co concentration governs the surface morphology of CoFe microstructures. Distinct morphological transitions are observed with the Co concentration from 0.025 to 0.15 M. Initially, at lowest Co concentration (0.025 M), the irregular, rough, and aggregated surface morphology was observed in CoFe-1(Fig. 1c). Further, increase in the Co concentration to 0.05 M emergence of more defined, rectangular-like structures, indicating the crystal growth of CoFe-2 (Fig. 1d). The pronounced transition towards well-defined hexagonal architectures upon increasing the dopant concentration (0.1 M) was observed in Fig. 1e, suggesting enhanced crystallographic order in CoFe-3. Afterwards, excess in Co concentration (0.15 M) starts to reduce the surface area of the NPs due to agglomeration (Fig. 1f). Collectively, the XRD, and FESEM indicate that CoFe-3 represents the optimum Co concentration; therefore, a systematic and thorough investigation should be required.

The TEM analysis was employed to investigate an in-depth assessment of structural and morphological features. The well-dispersed nanoscale morphology of the CoFe-3 has been displayed in Fig. 2a-d. Fig. 2a and b illustrate the TEM and HRTEM micrographs of CoFe-3 with a highly crystalline nature. The random distribution and moderate agglomeration of the sample occurred due to the effect of the calcination temperature^23^. The lattice fringe spacing observed in the HRTEM image of CoFe-3 NCs is 0.147 nm, which is indexed to (440) cubic crystallographic plane of the XRD. Such an alignment of HRTEM and XRD findings further validates excellent crystallinity and phase purity of the NCs. Furthermore, the average particle size of the sample was determined using a particle size distribution histogram (Fig. 2c). According to this study, the sample indicates an average particle size of 7–12 nm. Fig. 2d demonstrates the SAED pattern of the CoFe-3 consists of multiple diffraction rings. The obtained fringes in the SAED pattern corroborated with the lattice spacing and d-spacing values (400), (422), (511), and (440) of the XRD, which suggests the phase formation and purity of the CoFe-3.

Further, the EDS helps to investigate the elemental composition of the CoFe NCs. The presence of elements during mapping was depicted in Fig. 2e-h. From Fig. S1, it can be seen that 17.0%, 29.9%, and 53.1% of Co, Fe, and O elements have been traced in the CoFe-3. The presence of greater elemental composition of O than Co and Fe confirms the success of the synthesis protocol^13^. Additionally, the homogenous distribution of the Fe, Co, and O is being observed in the fabricated sample.

**Fig. 3:**
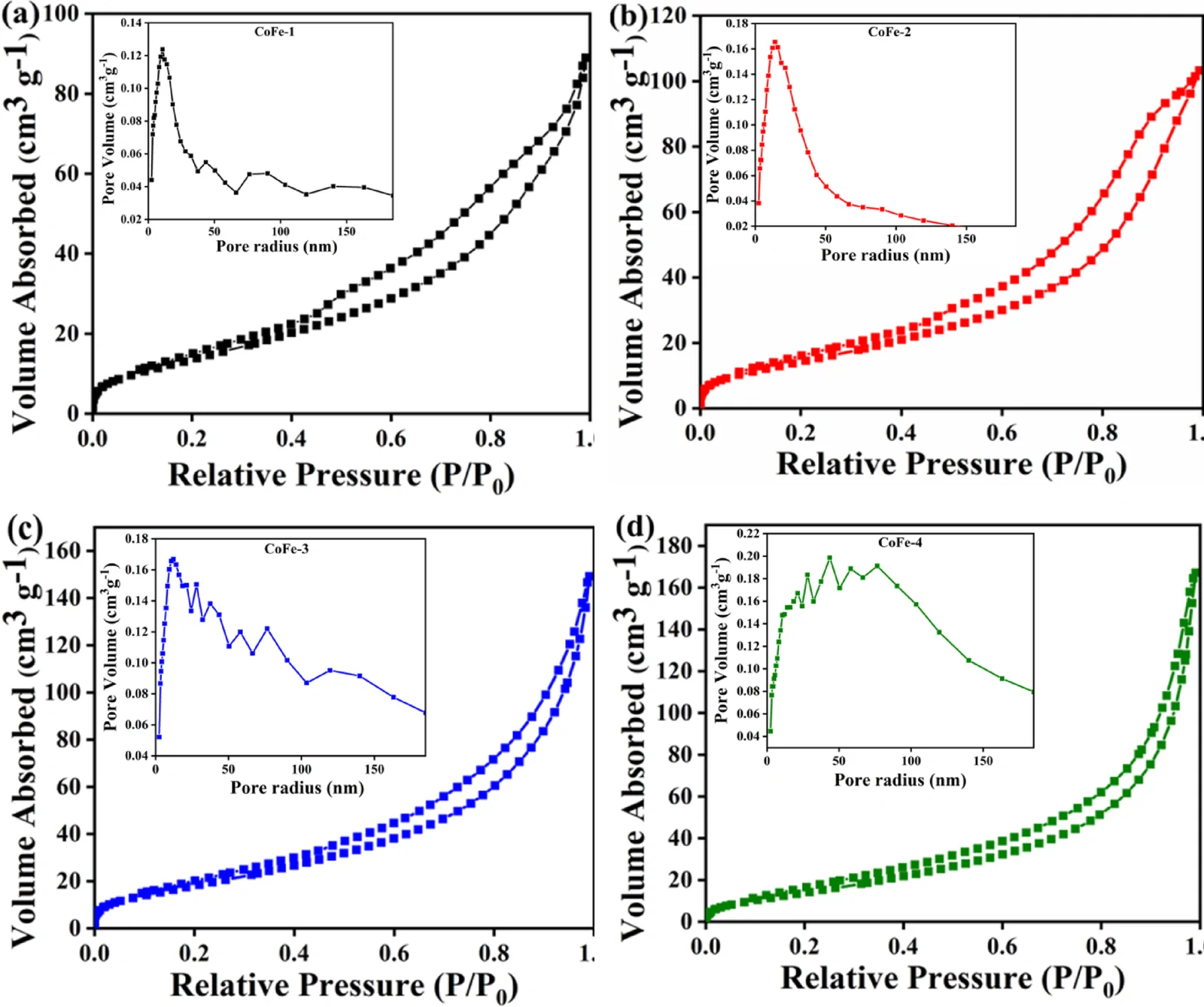
(**a**), (**b**), (**c**), (**d**) BET surface area analysis by N_2_ adsorption-desorption isotherm profile of CoFe-1, CoFe-2, CoFe-3, CoFe-4, along with the inset representing BJH pore size distribution.

To multipoint nitrogen adsorption-desorption measurements were performed for CoFe-1 to CoFe-4 batches (Fig. 3a-d) to evaluate their surface area and textural parameter. A larger surface area enhances the active sites availability, improves the adsorption behaviour, electron movement and accelerates the reaction rates, which ultimately strengthens the multifunctionality of the NCs^46^. The 56.93, 58.67, 74.98, and 63.01 m^2^/g surface area was calculated by BET analysis of the nanocomposites CoFe-1, CoFe-2, CoFe-3, and CoFe-4, respectively. These findings suggests that the CoFe-3 batch represents a remarkably large surface area compared to other NCs. The insets of Fig. 3 display the pore size distribution for the CoFe-1 to CoFe-4. The evaluation indicates a more promising pore size for CoFe-3 (16.17 nm), while the remaining three batches (CoFe-1, CoFe-2, and CoFe-4) exhibit pore sizes of 9.52, 10.77, and 12.77 nm, respectively. Such higher values of the relative surface area and pore size reinforces the magnetic behavior of the NCs^17^. Expanded surface area of the CoFe-3 enables the additional active sites, boosts the dye adsorption capacity and production of reactive species, which collectively improve the dye degradation rate. Additionally, it promotes the stronger photocatalyst-pollutant interaction^47^. While the proper pore size distribution enhances the mass transfer and diffusion of the reactant and product species, which are vital for photocatalysis. Simultaneously, porous framework improves the light utilization by inducing multiple internal reflections^48^. Along with dye degradation, higher surface area not only strengthens the charge accumulation capacity of the supercapacitor^49^ but also enhances the interaction of NCs with fungal cells during the antifungal activity, thereby disrupting the fungal membrane^50^. Combined XRD, FESEM, TEM and BET analysis of CoFe NCs, collectively reveals their phases, tunable morphologies, and surface area that plays crucial role in broad range of applications.

**Fig. 4:**
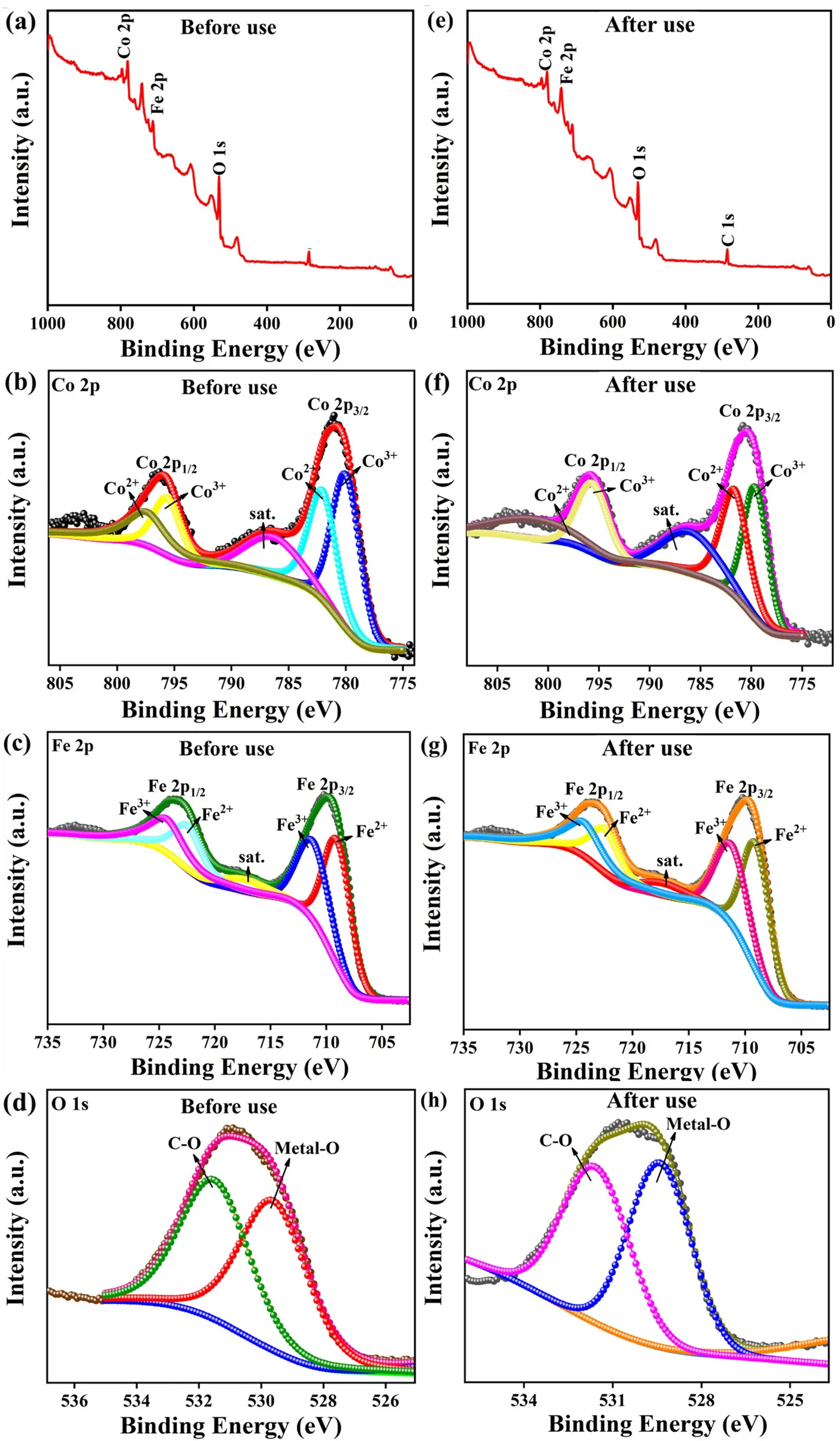
(**a**, **e**) XPS survey spectra illustrating elemental composition before and after its catalytic use, (**b**-**d**) deconvoluted Co 2p, Fe 2p, and O 1 s spectra of the catalyst before its use; and (**f**-**h**) corresponding spectra of the catalyst after its use, depicting surface chemical states.

XPS of CoFe-3 NCs before (Fig. 4a-d) and after (Fig. 4e-h) its catalytic use was utilizes to get deeper insight into the elemental composition and oxidation states^51^. As shown in Fig. 4a, the XPS wide scan spectrum indicates the presence Co, Fe, and O with characteristics peak at 797.99 eV, 714.31 eV, and 531.23 eV assigned to Co 2p, Fe 2p and O1s orbitals, respectively. The high-resolution XPS spectrum of Co 2p orbital (Fig. 4b) displays two prominent peaks at 781.01 eV and 796.17 eV, which are attributed to the Co 2p_3/2_ and Co 2p_1/2_ states. The Co 2p_3/2_ and Co 2p_1/2_ spectrum, upon deconvolution indicates the Co^2+^ at 782.16 eV and 797.62 eV, whereas peaks at 78.82 and 795.75 eV contributes to the Co^3+^. The satellite peak at binding energy 786.83 eV arising from the secondary photoelectron transition^52^. The Fe 2p spectrum (Fig. 4c) reveals two prominent peaks at 709.23 eV and 722.65 eV, representing Fe 2p_3/2_ and Fe 2p_1/2_ orbitals of the bivalent iron. The additional peaks at 711.39 eV and 724.50 eV corresponds to Fe^3+^, resulting Fe 2p_3/2_ and Fe 2p_1/2_ states. The presence of Fe^3+^ species further supported by satellite peak 717.26 eV, originated from shake-up phenomenon during photoelectron ejection^53^. The O 1 s spectrum exhibits two peaks, as depicted in Fig. 4d. The peak centred near 529.66 eV assigned to lattice oxygen associated with metal-oxygen bond such as Fe-O or Co-O in CoFe_2_O_4_. The signal at 531.60 eV indicate the oxygen functionalities linked to carbon atom in the metal oxide material^34^. Aforementioned findings revealed that the before use XPS spectra indicates well defined peaks reflecting to the material’s inherent chemical states. Furthermore, the post use XPS analysis (Fig. 4e-f) exhibits slight shift and intensity variation, particularly in the Co 2p states. This negligible change occurred due to the surface interaction between material CoFe-3 and reactant dye^54^^,55^.

**Fig. 5:**
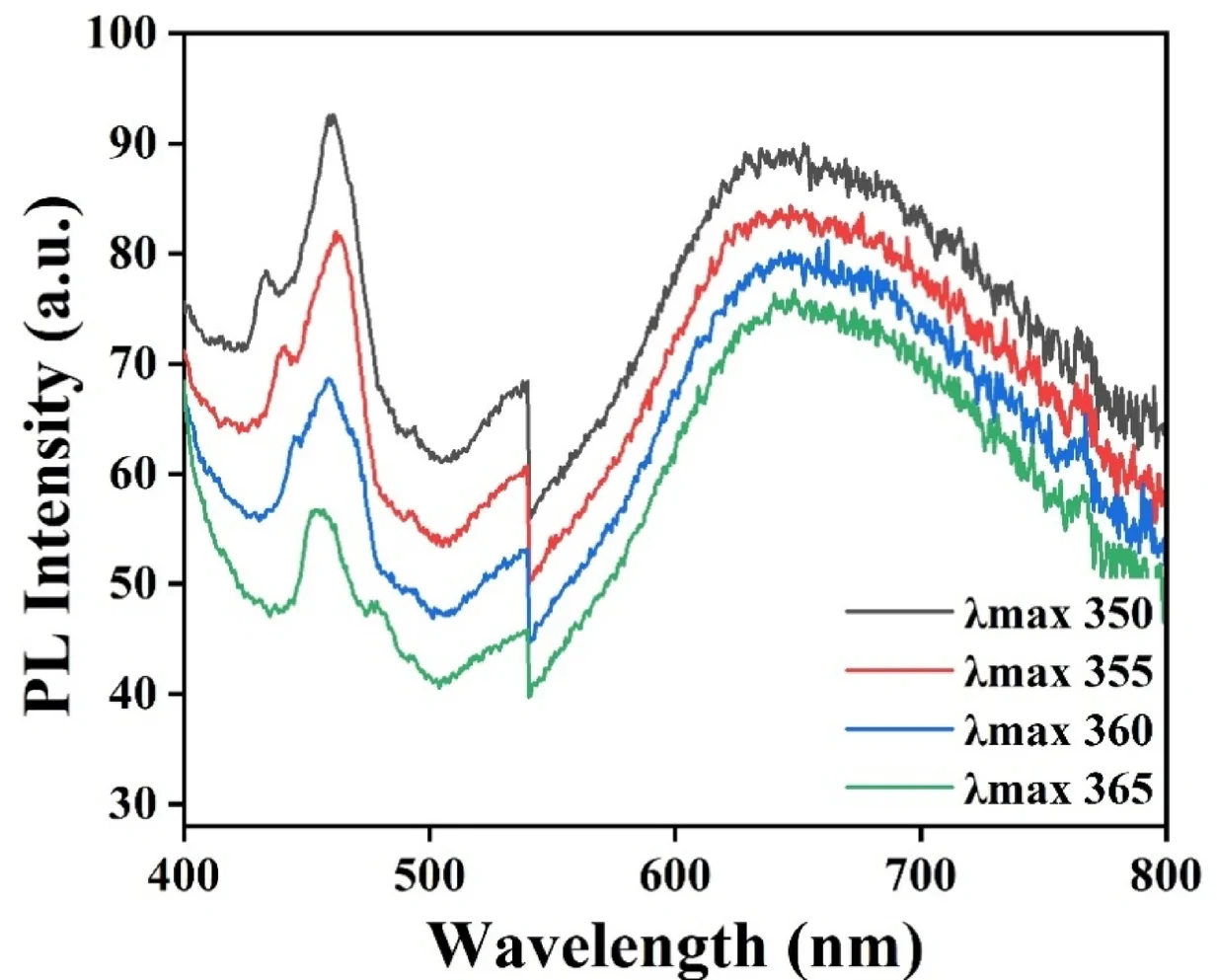
PL emission spectroscopy of CoFe-3 with the excitation maxima of 350, 355, 360 and 365 nm.

Photoluminescence spectroscopy (PL) was carried out to explore the electronic structure and charge carrier dynamics of CoFe-3 at ambient temperatures. PL measurements were measured between 400 and 800 nm with excitation wavelengths of 350, 355, 360, 365 nm, as shown in Fig. 5. Upon excitation of 350 nm, the CoFe-3 displayed intrinsic emission peak around 461 and 642 nm. The emission peak at 461 nm is located in the blue region of the visible spectrum and typically linked to the surface defect or vacancy related states^56^. In contrast, the peak near 642 nm appears in the red region and is generally associated to deep level defects or d-d electronic transition of Co^2+^/Fe^3+^^57^. A noticeable reduction in the PL intensity with minor shift in the emission peaks occurs as we change the excitation wavelength from 355 to 365 nm. Such an excitation dependent trend indicates the several energy level inside the bandgap^53^. Meanwhile the declined peak intensity at higher excitation wavelengths suggests more efficient charge separation and suppressed radiative recombination, favoring the photocatalytic activity^53^. Furthermore, the reduced peak intensity indicates the presence of defect states, particularly oxygen vacancies within the material. Such a vacancies acts as an effective charge trap, promoting electron-hole recombination and thereby boosting the ROS generation. In agreement with this, Lu et al. reported efficient charge transfer engineering which improves electron-hole separation and suppresses recombination, resulting the superior photoreduction performance^58^.

### Dye degradation by photo-Fenton process

**Fig. 6:**
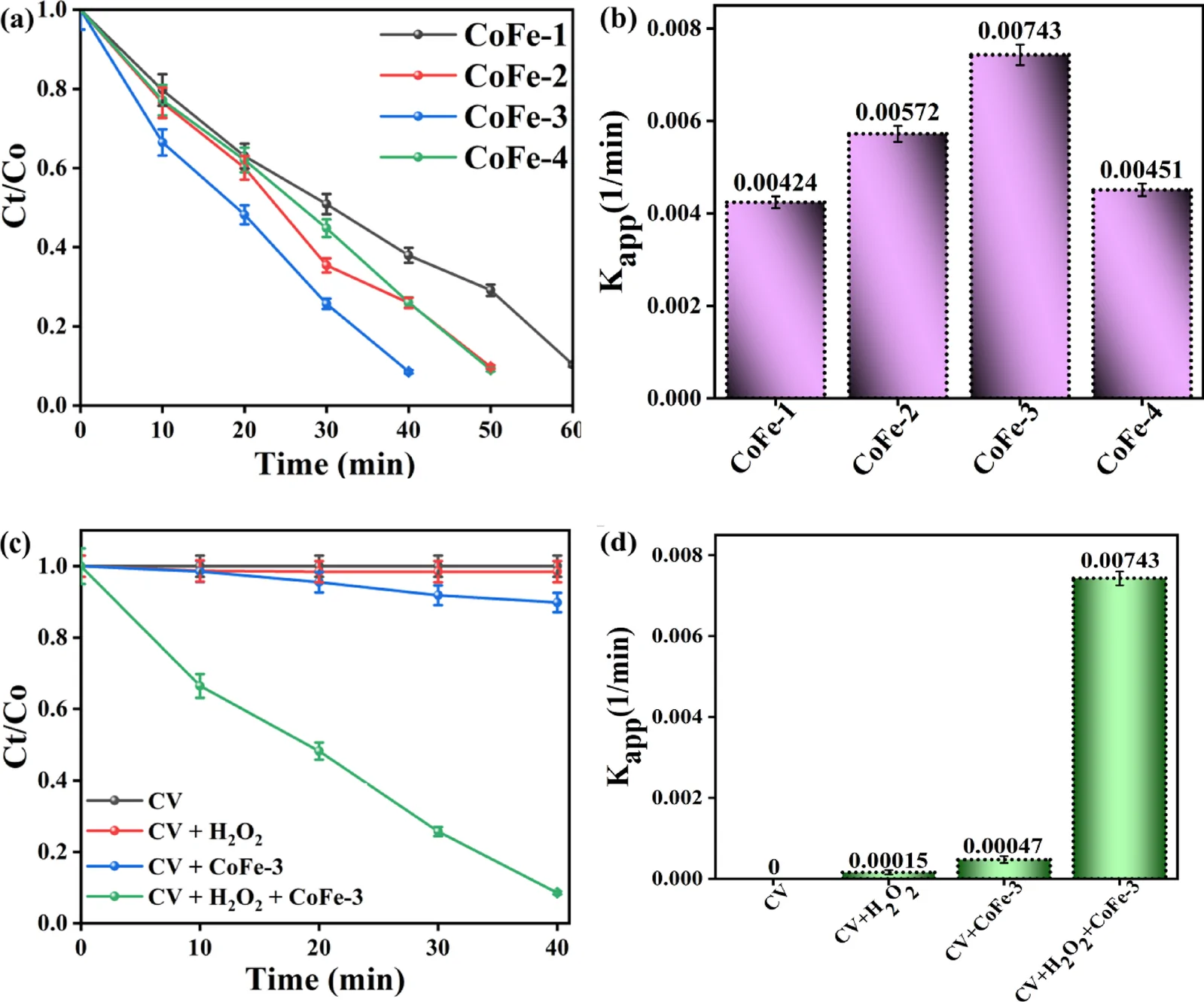
(**a**) and (**b**) photo-Fenton activity of a series of cobalt ferrite batches (CoFe-1 to CoFe-4) and corresponding synergy effects; (**c**) and (**d**) Optimal experimental parameters influencing the CV degradation along with their synergy effect.

At first, the photocatalytic degradation capacity of the series of CoFe batches was investigated on crystal violet (CV) as a model dye pollutant. The influence of the CoFe-1, CoFe-2, CoFe-3, and CoFe-4 on CV degradation was elucidated by the photo-Fenton process and their corresponding synergy effect has been shown in Fig. 6a and b. A progressive enhancement in degradation efficiency was observed from CoFe-1, CoFe-2, and CoFe-3; however, CoFe-4 reduces the rate of the degradation reaction. Specifically, CoFe-1, CoFe-2, CoFe-3, and CoFe-4 achieve 89.64%, 90.26%, 91.44%, and 90.90% degradation efficiency, requiring 60, 50, 40, and 50 min., respectively. The CoFe-3 exhibits the highest degradation efficiency within a minimum time, confirming it as an optimal catalyst composite. XRD analysis reveals the phase shift from Co doped Fe_3_O_4_ to pure CoFe_2_O_4_ substantially elevates the catalytic activity of CoFe-3; as it improves the charge separation, magnetic recovery and visible light sensitivity. The formation of well-organized hexagonal structures was portrayed by the FESEM images. Such a structures enlarges the surface area and exposes more active facets, thereby enhancing dye adsorption, promoting ROS production and redox activity of the catalyst. While, further higher surface area improves adsorption capacity and charge accumulation ability of the CoFe-3. This outperforming behavior of CoFe-3 because of its optimal Co concentration possesses best combination of crystallinity, morphology, ROS production, charge accumulation and an active site availability. Therefore, the CoFe-3 batch was further used for a detailed study of the photo-Fenton reaction, supercapacitor performance and antifungal activity.

The photo-Fenton activity of the CoFe-3 along with its synergy effect was suggested by testing its activity under different reaction condition such as CV, CV + H_2_O_2_, CV + CoFe-3, CV + H_2_O_2_ + CoFe-3 and their results are presented in a Fig. 6c and d. In these proposed experiments, no significant degradation occurred in CV, and CV + H_2_O_2_ even in presence of light. However, introduction of CoFe-3 in the CV + CoFe-3 initiates the CV degradation in the presence of light penetration, resulting in a photocatalytic reaction^11^^,59^. Whereas, the full photo-Fenton system (CV + H_2_O_2_ + CoFe-3) produces a remarkable enhancement in CV degradation rate up to 91.44%, confirming the strong interaction among H_2_O_2_, CoFe-3, and light. The performance of the existing catalyst is compared with the earlier reports in Table S1, showcasing favorable degradation capability of the current material. Based on these findings, the corresponding degradation mechanism^60^ are outlined below;

6 $$\:{CoFe}_{2}{O}_{4\:}+hv\:\to\:\:{CoFe}_{2}{O}_{4\:}({h}^{+}+\:{e}^{-})$$

7 $$\:{CoFe}_{2}{O}_{4\:}\left({e}^{-}\right)+\:{H}_{2}{O}_{2}\to\:\:{CoFe}_{2}{O}_{4\:}+\:{OH}^{-}+\:\bullet\:OH\:$$

8 $$\:{CoFe}_{2}{O}_{4\:}\left({h}^{+}\right)+\:{OH}^{-}\to\:\:{CoFe}_{2}{O}_{4\:}+\:\:\bullet\:OH\:$$

9 $$\:CV+\:\bullet\:OH\to\:\:Degraded\: product\:$$

Under light illumination, initially catalyst starts to produce photoinduced $\:{h}^{+}/{e}^{-}$ pairs, as represented in Eq. (6). Afterwards the photoexcited $\:{e}^{-}$ on the CoFe interacts with H_2_O_2_, generating hydroxide ions $\:\left({OH}^{-}\right)\:$and hydroxyl radical $\:\left(\bullet\:OH\right)$ as shown in Eq. (7). Meanwhile, $\:{h}^{+}$ formed upon the light penetration, oxidize the $\:{OH}^{-}$ and convert it to $\:\bullet\:OH\:$ (Eq. (8)). As a result, the generated $\:\bullet\:OH\:$ radical attacks dye molecule causing their degradation (Eq. (9)).

### Determination of pHzpc

The mechanism between the adsorbent (CoFe NCs) and the adsorbate was explored by performing a zero-point pH study (Fig. S2), by utilizing the salt addition method. Initially, the pH of the solution was adjusted to 2, 4, 6, 8, and 10 by adding 0.1 N HCl/NaOH to a series of Erlenmeyer flasks containing 20 mL of the NaNO_3_ solution. Then, this flask was rotated overnight at 130 rpm under ambient temperature in a shaker incubator. At last, the final pH (pH_f_) of the solution was examined and further used to calculate the ΔpH (Eq. 10).

10 $$(\Delta pH{\rm{ }} = {\rm{ }}pHf{\rm{ }} - {\rm{ }}pHi)$$

Aforementioned Eq. (10) was used to determine ΔpH; further, these values were plotted against the initial pH (pH_i_) of the solution. Lastly, the intersecting point on the plotted graph was evaluated as the pHzpc of the CoFe NCs. This intersection point yields a value of ΔpH as 6.35. The outcome demonstrates the acidic properties of the catalyst. When the pH of reaction mixture is higher than 6.35, the surface hydroxyl group undergoes deprotonation (Eq. 11), producing a net negative charge^61^. This negatively charged surface promotes the cationic adsorption, subsequently enhancing the cationic dye degradation^62^. As a result, the synthesized catalyst CoFe-3 demonstrates the notable degradation of the cationic dye CV. Similar findings of the catalyst related to the adsorption mechanism of the cationic (pH> pHzpc) as well as anionic (pH< pHzpc) dyes were reported earlier^13^^,63^. Consequently, such a condition also enhances the electrostatic affinity of the CoFe and accelerates the fast Co^2+^/Co^3+^and Fe^2+^/Fe^3+^ redox cycling. It strengthens the H_2_O_2_ activation and promotes the generation of $\:\bullet\:OH\:$radical, ultimately elevates the catalytic activity of CoFe-3^63^.

11 $$\:\equiv\:M-OH\:\to\:\:\equiv\:M-O\:+\:{H}^{+}$$

### The correlation between catalyst dosage and degradation efficiency

**Fig. 7:**
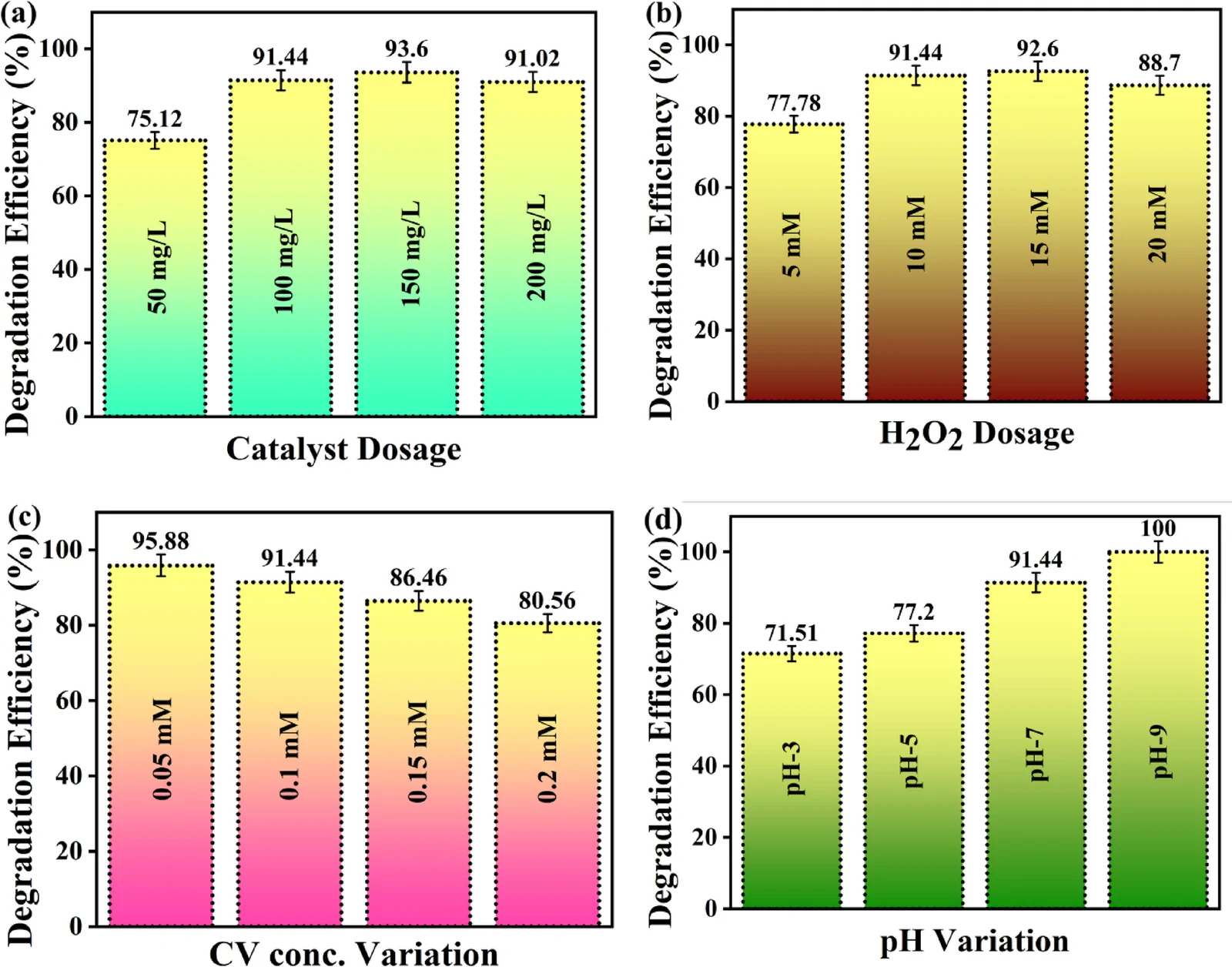
The influence of varying parameters on the degradation efficacy of the CV; (**a**) Catalyst Dosage (dye: 0.1 mM, pH: 7; H_2_O_2_:10 mM), (**b**) H_2_O_2_ Dosage (dye: 0.1 mM; pH: 7; catalyst (CoFe-3): 100 mg/L), (**c**) Initial CV concentration (pH: 7; H_2_O_2_:10 mM; catalyst (CoFe-3): 100 mg/L), (**d**) Initial pH of the solution (dye: 0.1 mM; H_2_O_2_:10 mM; catalyst (CoFe-3): 100 mg/L).

Catalyst loading is a crucial factor affecting the dye degradation, therefore optimizing catalyst dosage is essential to prevent excessive or inefficient catalyst utilization during reaction. Thus, the effect of catalyst dosage, ranging from 50 mg/L to 200 mg/L, was examined in this study and is elucidated in Fig. 7a. The catalyst exhibited efficiency of 75.12% at 50 mg/L, which reached 93.60% at 150 mg/L of catalyst. Beyond this, no significant change was observed at higher initial CV concentration. These findings show a close similarity to other catalytic studies^64^^,65^. This observed trend in the results was explained by the following reasons: i)The photocatalytic activity of the catalyst was minimum when the catalyst load was minor; as the smaller number of photons was absorbed by the catalyst^66^. ii) As the amount of catalyst increased at 100 and 150 mg/L, it provides an increased number of active surface area, which acts as center for absorption of the photons. Thus, an increase in the dye degradation percentage was observed at this catalyst dosage^66^. iii) Further increase in the catalyst load, i.e., 200 mg/L, shows the saturation effect and increases the opacity of the solution, which inhibits the light penetration^66^^,67^. As a result, the catalyst wastage occurred because no significant change in the initial dye concentration was achieved.

### The correlation between H_2_O_2_ dosage and degradation efficiency

The H_2_O_2_ is an oxidizing agent that upsurges the generation of the hydroxyl radical; thus, H_2_O_2_ leads the photo-Fenton process by increasing the rate of reaction. Therefore, it is necessary to carefully study the optimization of the H_2_O_2_ doses. Here, the influence of the 5 mM to 20 mM H_2_O_2_ dosage on CV degradation is evaluated and depicted in Fig. 7b. No appreciable change in the degradation percentage was observed when the H_2_O_2_ dose was 5 mM. At doses of 10 and 15 mM H_2_O_2_, the degradation percentages reached 91.44% and 92.60%, respectively. At 10 and 15 mM H_2_O_2_, dose amounts of H_2_O_2_ are relatively high; as a result, a larger number of hydroxyl radical was generated, which enhances the rate of dye degradation^68^. Furthermore, the additional increase in the H_2_O_2_ dose to 20 mM results in a decline in the dye degradation percentage. The excessive addition of the H_2_O_2_ starts to inhibit the generation of hydroxyl radical due to its scavenging effect^68,69^–^70^. Due to these observed consequences, we used the 10 mM H_2_O_2_ dose as an optimum parameter for dye degradation.

### The correlation between CV concentration and degradation efficiency

The initial concentration of the dye contaminant has a remarkably significant effect on the degradation efficiency. The relationship between the initial dye concentration (0.05 mM to 0.2 mM) with the degradation efficacy of the catalyst was illustrated in Fig. 7c. More than 90% of the CV was successfully degraded when the initial CV concentration was 0.05 mM and 0.1 mM; it started to drop down to 86.46% and 80.56% as the concentration of CV increased from 0.15 mM to 0.2 mM. Increasing the initial dye concentration further increases the number of dye molecules in the solution. The excess of dye molecules adsorbs onto the active site, and restricts the penetration of light on the catalyst’s surface which ultimately affects the generation of hydroxyl radicals, thereby reducing the rate of the degradation reaction^67^. Omer et al^64^. reported similar findings for the dye degradation with a change in initial dye concentration. Their results depict the 97.14% and 96.74% degradation of CV and MB with 40 mg/L of initial dye concentration. Further, this dye degradation fell to 89.1% and 90.4% as the initial concentration of the dye was raised to 100 mg/L^64^.

### The correlation between pH and degradation efficiency

The pH is an important monitoring parameter of AOPs and has a considerable effect on the dye degradation reaction. The impact of a 3 to 9 pH range on the photocatalytic degradation of the dye by CoFe-3 catalyst was studied in the current work, and the results have been reported in Fig. 7d. The alteration in the pH from acidic to alkaline shows a rise in the dye degradation percentage. The surplus amount of protons is present when the pH of the solution is acidic; therefore, the active sites of the catalyst become protonated^64^. This protonation reduces the degradation percentage at acidic pH. The number of negative charges on the catalyst increases during alkaline conditions; therefore, the cationic dye CV gets easily adsorbed on the surface of the catalyst. At a higher pH range, the noteworthy electrostatic interaction between the cationic dye CV and the negatively charged surface of the catalyst occurred, which increases its degradation rate^64^. The 100% dye degradation was observed at alkaline pH-9. The results of pH-9 were ignored even after their heightened degradation efficiency; it happens because the violet colored CV solution drastically changed to colorless after the addition of hydrogen peroxide^65^. Sumitha et al. also reported such an ignored degradation result of crystal violet under an alkaline environment, i.e., pH 11^65^. According to the above-mentioned results the pH 7 was used as an optimal parameter in the current work for the CV degradation^64^^,71^.

### Radical scavenger study

To understand the dye degradation mechanism, radical inhibition studies were performed to evaluate the significance of active radicals (•O_2_^−^ and •OH). The trapping agents including isopropanol, n-Butanol and p-Benzoquinone were employed to identify the dominant reactive species involved in the photodegradation process. The •OH radical were efficiently suppressed by scavenger n-Butanol and isopropanol^3^, while p-Benzoquinone selectively quenches the •O_2_^−^ radical^72^. The impact of scavenger on the degradation behaviour is illustrated in the Fig. S3. The incorporation of the scavenger’s isopropanol, n-Butanol and p-Benzoquinone suppresses the degradation efficiency, which led to drastic drop in the degradation efficiency from 91.44% to 11.66%, 14.12%, and 43.64%, respectively. According to the results, the extent of degradation suppression can be arranged as: n-Butanol ˃ isopropanol ˃ p-Benzoquinone ˃ without scavenger. In the reaction system, the activity of free •OH radical is inhibited by the n-Butanol^73^, whereas the isopropanol targets the catalyst bound •OH radical^74^. The scavenger p-Benzoquinone act as •O_2_^−^ radical scavenger^53^. Compared to n-Butanol and isopropanol, p-Benzoquinone showed relatively smaller effect on the inhibitory effect on the dye degradation process. This investigation indicates, •OH radical as a main contributor; while •O_2_^−^ radical serves as a secondary contributor during the degradation reaction.

### Anti-interference ability

Complex ionic constitution in may limit the practical applicability of the catalyst. Therefore, the anti-interference ability of CoFe-3 was evaluated in presence of common ions i.e. Cl^−^ and HCO_3_^−^ and practical water test. And their results were reported in the Fig. S4. The presence of Cl^−^ ions significantly affects the catalytic performance as compare to HCO_3_^−^ ions. This behaviour is likely due to preferential adsorption of Cl^−^ ions on the catalyst surface which compete with the dye molecule for reactive species, thereby suppressing oxidative degradation^72^. Additionally, dye degradation performance was tested in tap and lake water to assess the stability of the catalyst in the realistic conditions. Notably, dye degradation efficiencies showed 89.04 and 87.87% in tap and lake water, respectively. The negligible influence of both co-existing ions and practical water confirms the anti-inference ability of CoFe-3 catalyst.

### Reusability

**Fig. 8:**
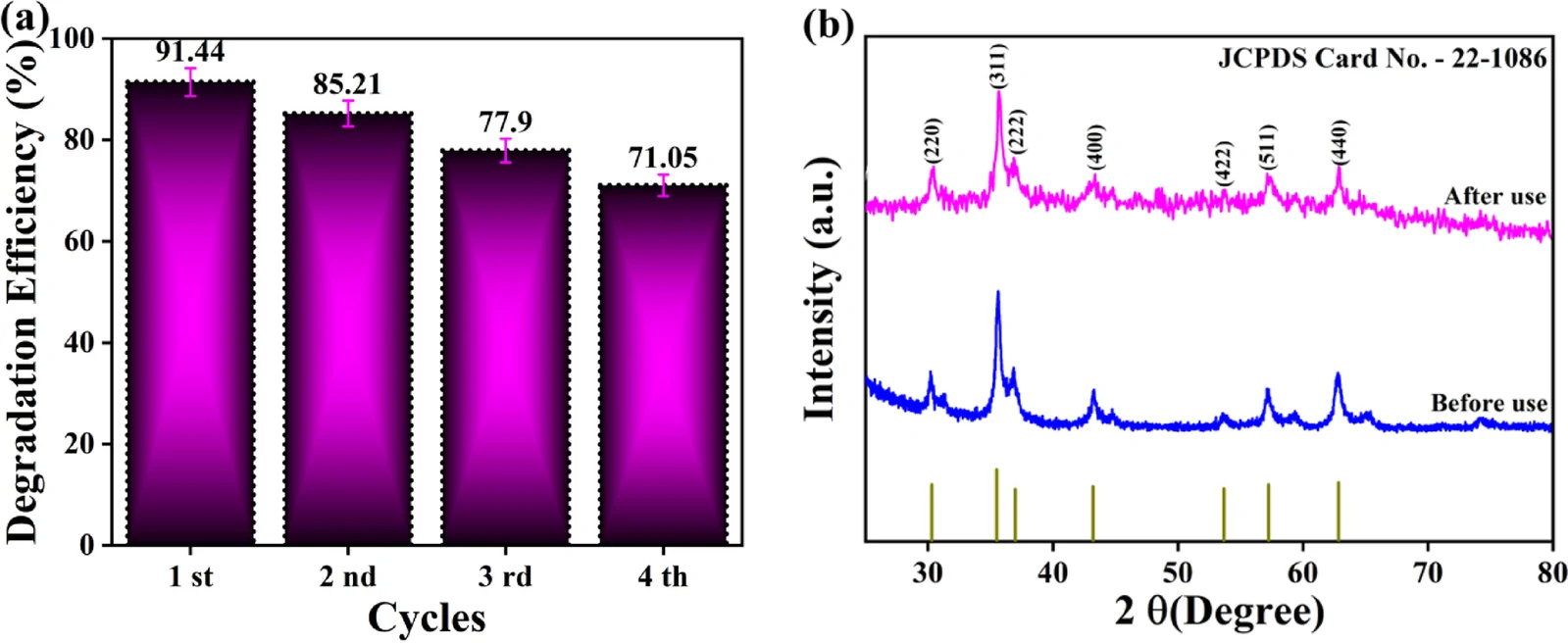
(**a**) Recycling performance of CoFe-3 for CV degradation and (**b**) XRD analysis of photocatalyst CoFe-3 before and after catalytic use.

For the practical process, the performance of the catalyst depends on its reuse ability. The best batch CoFe-3 was repeatedly used 4 times in this study, and its results have been illustrated in Fig. 8a. A slight reduction in the degradation percentage was observed even after 4 consecutive cycles. Initially, 91.44% of the dye was degraded in its 1 st use, while this degradation rate changed to 85.21%, 77.90%, and 71.05% upon its 2nd, 3rd, and 4th cycles. Hence, this result indicates the better reliability and beneficial environmental applicability of the catalyst to treat the wastewater. Furthermore, the XRD investigation of CoFe-3 before and after its catalytic use was examined, as presented in Fig. 8b. These results suggest the minor changes in the peak intensity after five successive cycles. These findings indicate the good structural stability of the material with no phase transformation occurring during the repeated usage.

### Electrochemical application

**Fig. 9:**
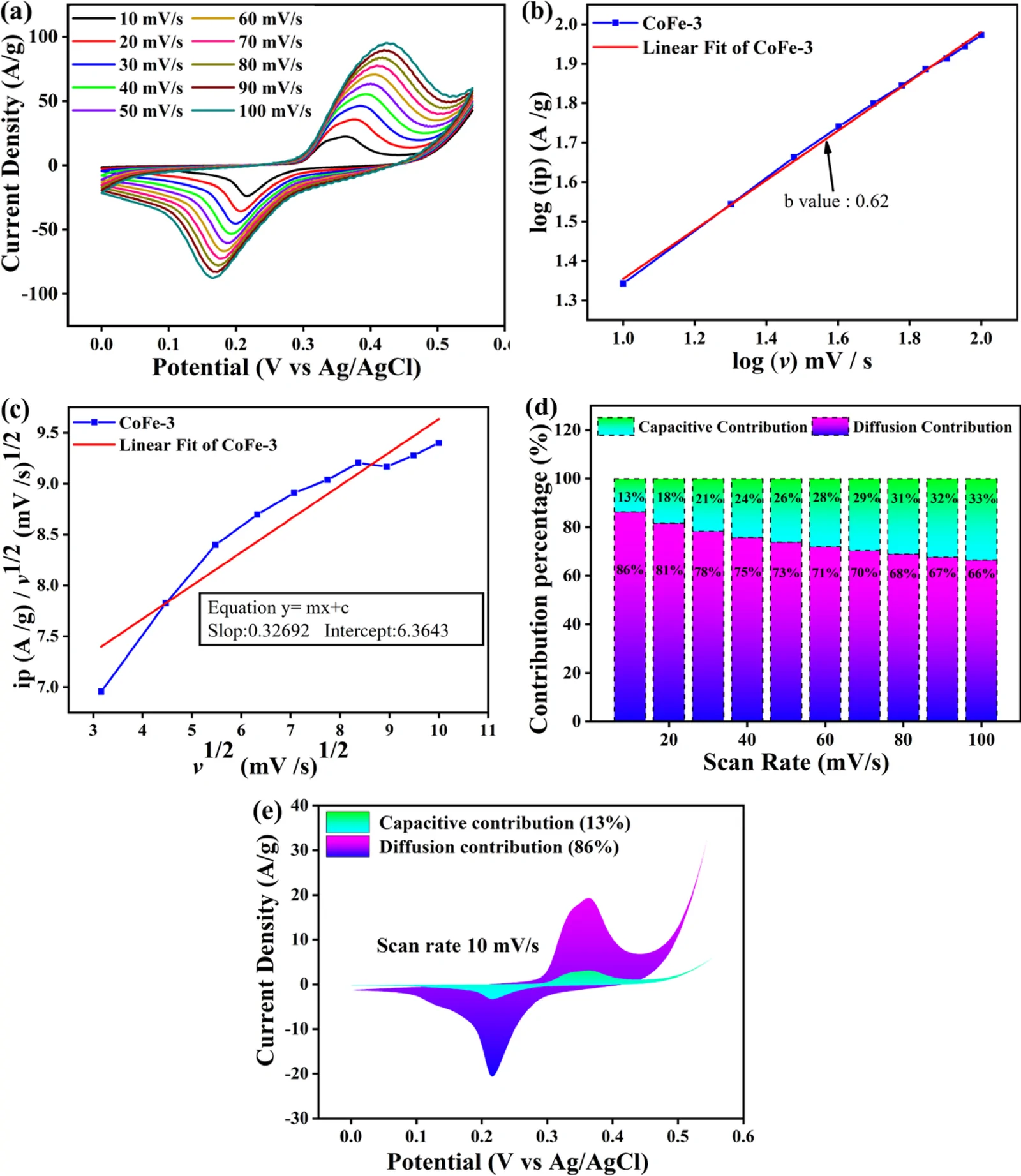
(**a**) CV curves of CoFe-3 obtained at scan rates between 10 and 100 mV s⁻¹; (**b**) Logarithmic relationship between scan rate and current for calculation of the b-value; (**c**) The plot of square root of scan rate vs. anodic current/square root of scan rate showing the fitted slope and intercept; (**d**) Comparative bar diagram of capacitive and diffusion-controlled contributions at various scan rates; and (**e**) Capacitive- and diffusion-dominated charge storage contributions of CoFe-3 at 10 mV s⁻¹.

CoFe-3 was chosen for supercapacitor application because its higher surface area, improved conductivity, and abundant Fe/Co redox-active sites enable efficient ion diffusion and enhanced charge storage. The cyclic voltammetry profiles, displayed in Fig. 9a, were recorded at scan rates ranging from 10 mV/s to 100 mV/s. For CoFe-3, the potential window was determined to be 0 V to 0.55 V. Notably, all CV loops retained their shape without distortion as the scan rate increased, indicating excellent surface capacitive behavior across the electrodes. The CV curves showed distinct redox peaks, confirming that CoFe-3 electrodes’ function as battery-type supercapacitive materials due to the presence of faradaic reactions. This performance was evaluated in a 2 M KOH electrolyte^75^ (pH ≈ 14), where high OH concentration facilitates a reversible redox process at the Co and Fe sites. The superior mobility of K^+^ ions minimizes the internal resistance, insuring rapid charge transfer kinetics and efficient ion diffusion. The specific capacitance values for the CoFe-3 electrode were calculated using Eq. (12), resulting in a maximum capacitance of 214.11 F/g at a scan rate of 10 mV/s.

12 $$\:Cp=\frac{\int\:I\:dV}{2mv\varDelta\:V\:}\:\:\:F/g$$

In this context, ‘Cp’ represents the specific capacitance measured in F/g, while ʃ IdV denotes the integrated area under the CV curve, ‘m’ indicates the active material mass (g), ‘v’ refers to the scan rate in mV/s, and ‘ΔV’ signifies the potential window (V). A decrease in capacitance values is observed as the scan rate increases. At lower scan rates, electrolytic ions have sufficient time to diffuse into the pores of the active material, enhancing specific capacitance. Conversely, at higher scan rates, the capacitance decreases because electrolytic ions primarily interact with the electrode surface without effectively penetrating the internal pores. This behaviour is likely attributed to the limited diffusion of ions, which restricts their access to deeper pores within the electrode structure at high scan rates^76^. To understand the enhanced electrochemical performance of the optimized CoFe-3 electrode, the electrochemically active surface area (ECSA) was evaluated. The double-layer capacitance (Cdl), obtained from CV curves in the non-Faradic region (Fig. S5(a)) and the corresponding current density versus scan rate plot (Fig. S5(b)), was calculated to be 0.18 mF. The ESCA was estimated using following Eq.,

13 $$\:ECSA=\:\frac{Cdl}{Cs}$$

Where, Cs = 0.040 mF cm^− 2^, the value reported for the alkaline KOH solution. The derived ECSA value 4.5 cm^2^. The increased ECSA indicates a higher number of accessible active sites, which facilitates efficient OH^−^ ion diffusion and better utilization of the Co/Fe spinel structure, resulting in enhanced electrochemical performance.

The Dunn power-law equation, presented in Eq. (14), was utilized to calculate the b-value (slope) from the CV spectra, providing insights into the charge storage behaviour of the CoFe-3 electrode.

14 $$\:{I}_{capacitive}+{I}_{diffusion\:}=a{v}^{b}$$

Here, $\:{I}_{capacitive}$ and $\:{I}_{diffusion\:}$represent the contributions from surface-controlled and diffusion-controlled currents, respectively, with ‘*a*’ and ‘*b’* serving as adjustable parameters. The current response uses the capacitive method if ‘b’ is greater than or equal to 1.0. Conversely, it indicates that the involvement of both surface-controlled and diffusion-controlled mechanisms occurs when b values lie between 0.5 and 1^77^. In Fig. 9b, for CoFe-3 electrodes, the b values are found to be 0.62, indicating that diffusion-controlled processes overpower surface-controlled contributions. This diffusion-dominated contribution reflects battery-type behavior because its Co-rich spinal structure provides multivalent Fe and Co faradic redox-active sites, which undergo reversible redox reaction during the charge-discharge process^78^.

Moreover, the current response Eq. (15) can be divided into two parts, allowing the independent determination of the respective contributions of diffusion-controlled charge storage and surface capacitive processes.

15 $$\:ip=K1v+k2\surd\:v$$

As indicated by Eq. (15) the terms ‘k1v’ and ‘k2$\:\surd\:$v’ represents the capacitive and diffusion-controlled currents, respectively, as demonstrated in Fig. 9c. It is essential to highlight that reversible surface faradaic redox reactions, along with substantial surface polarization, lead to diffusion capacitance contributions. In a battery-type SCs, the movement of ions within the active electrode layer contributes more to the diffusion-controlled process, driven by the intercalation and de-intercalation of electrolytic ions. Figure 9c displays the relationship of the anodic peak current (i) and the square root of scan rate. The scan rate-dependent capacitive and diffusion contributions are illustrated as a bar diagram in Fig. 9d. CoFe-3 based SCs mostly rely on diffusion-controlled mechanisms (86%) as opposed to surface capacitive mechanisms (13%) at scan rate of 10 mV/s, as seen in Fig. 9e.

**Fig. 10:**
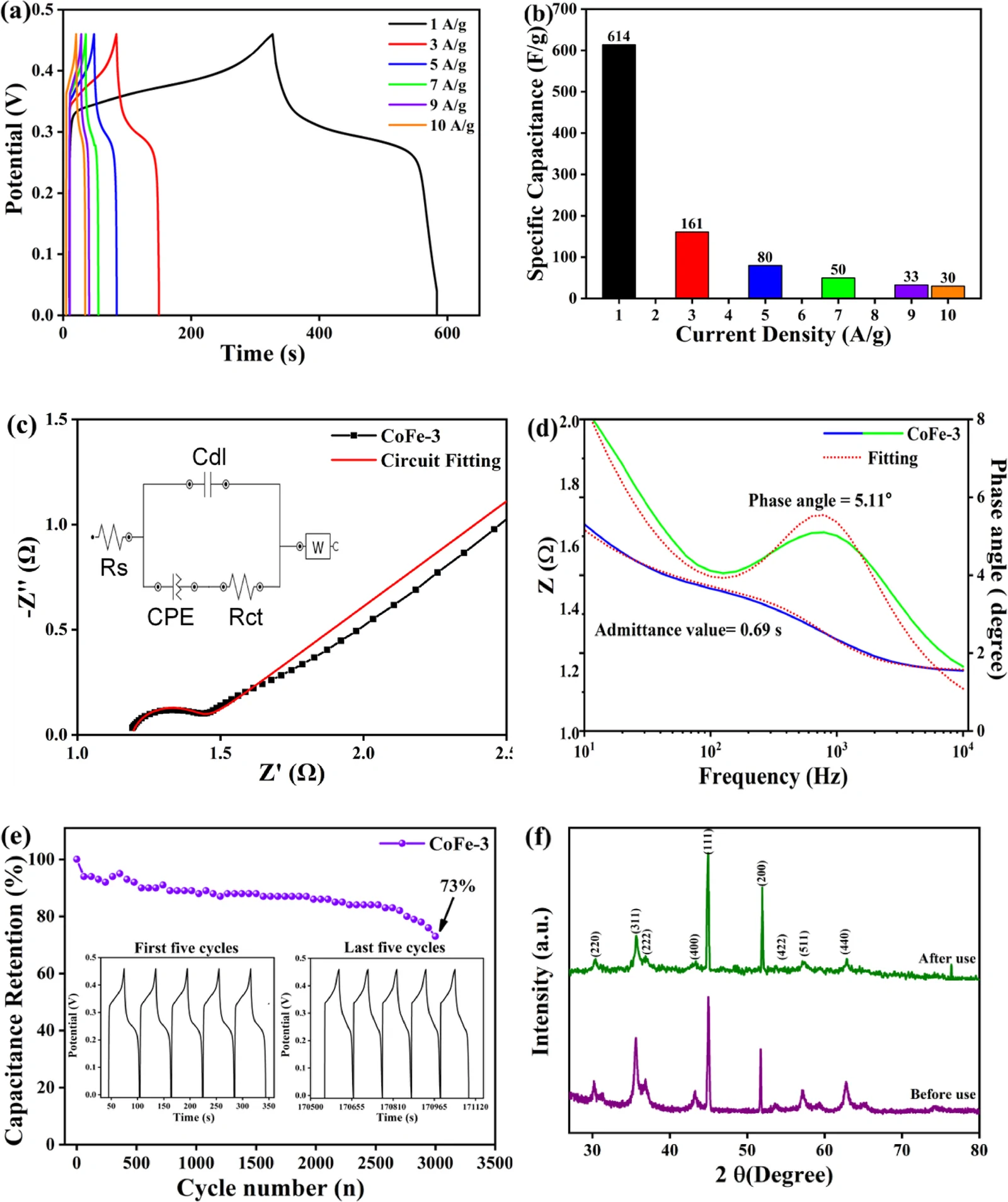
(**a**) GCD curves of CoFe-3; (**b**) Comparative plot of specific capacitance of CoFe-3 at 1, 3, 5, 7, 9 and 10 A/g current densities; (**c**) Nyquist plots of CoFe-3 electrode and inset shows the equivalent Randles circuit; (**d**) Bode plot of the CoFe-3 electrode; (**e**) GCD cyclic stability of CoFe-3 at 5 A/g current density, with the first and last five GCD cycles displayed in the inset; (**f**) XRD pattern of CoFe-3 before and after electrochemical stability.

To further investigate the electrochemical performance of CoFe-3, GCD curves at various current densities were tested. The GCD curves of CoFe-3 recorded at current densities of 1, 3, 5, 7, 9, and 10 A/g are shown in Fig. 10a. All the curves exhibit clear and well-defined distinct charging and discharging plateaus, indicating battery-type supercapacitor behaviour and effective utilization of the CoFe-3 electrode. The specific capacitance (Cs) was determined from the GCD curves using the following Eq. (16):

16 $$Cs=\:\frac{I\varDelta\:t}{m\varDelta\:v}$$

In this case ‘I’ denotes the discharge current, Δt represents the discharge time, and Δʋ symbolizes the potential window. The symbol ‘m’ refers to the mass of the active material^79^. The Cs values are found to be 614, 161, 85, 50, 33, and 30 F/g at current densities of 1, 3, 5, 7, 9, and 10 A/g, respectively. The Cs values of the CoFe-3 electrode at different current densities are summarized in a bar diagram in Fig. 10b The energy density and the corresponding power density of CoFe-3 at different current densities are calculated based on the following Eqs. (17) and (18):

17 $$E.D. (Wh/kg) =\:0.5\:\frac{Csp{\:(V2-V1)}^{2}}{3.6}$$

18 $$P.D (W/kg) =\:\frac{\:E.D}{\varDelta\:t}\:3600$$

where ‘(V2 – V1)^2’^ is the potential window, and ‘Δt’ is the discharge time^80^. Accordingly, the energy density of CoFe-3 is calculated as 15.04 Wh/kg, and the corresponding power density is obtained to be 217.16 W/kg at a current density of 1 A/g.

Measurements of EIS for the CoFe-3 electrode were conducted with a potential amplitude of 10 mV in the frequency range of 0.1 Hz to 10^5^ Hz (Fig. 10c). The measured R_s_ values are determined to be 1.19 Ω while charge transfer resistance (R_ct_) 0.20, indicated by the diameter of the semicircle formed by the Nyquist curve as it moves from the higher to the medium frequency zone. The reduced diameter of the semicircle indicates a low R_ct_ value, which facilitates charge transfer between the electrode and electrolyte, thereby enhancing the material’s electrochemical performance. The EIS data were fitted using an equivalent circuit (Inset), which matches well with the experimental results. The experimental and circuit-fitted values of R_ct_ and Rs for CoFe-3 are tabulated in Table 1.

**Table 1:** Experimental R_s_ and R_ct_ values of CoFe-3 electrodes.

Sr. No.	Materials	Experimental values		Circuit fitted values	
		*R*_s_ Ω	*R*_ct_ Ω	*R*_s_ Ω	*R*_ct_ Ω
1	CoFe-3	1.19	0.20	1.19	0.25

According to Randviir^81^,the heterogeneous electron transfer rate constant *k*° was calculated to further quantify redox kinetics by using the formula:

19 $$\:K^\circ\:=\frac{RT}{{n}^{2}{F}^{2}AC{R}_{ct}}$$

For the CoFe-3 electrode in 2 M KOH, the circuit fitted Rct 0.25 Ω obtained *k*° was 5.32 × 10^− 4^ cm s^− 1^ (*n* = 1). The *k*° value confirms the rapid electron transfer occurring at the electrode-electrolyte interface. In Fig. 10d, the Bode plot shows the impedance (Z) and phase angle of the CoFe-3-based SC as a function of applied signal frequency. The phase angle peaks at 5.11°, highlighting the material’s hybrid charge storage mechanism. An admittance value of 0.69 s indicates the material’s good capacitive performance, making it suitable for energy storage applications. These results indicate the potential of CoFe-3 based electrodes for battery-type SCs, offering low resistive losses and fast charge/discharge capabilities. The excellent electrochemical characteristics of CoFe-3 such as large specific capacitance and small charge transfer resistance are directly related to the optimized structural and electronic properties. This suggesting that compositional tuning improves the kinetics of electron transport and ion diffusion hence improving energy storage performance.

The electrochemical stability of the CoFe-3 electrode was tested for 3000 charge/discharge cycles at a current density of 5 A/g (Fig. 10e). GCD curves exhibit exceptional cycling stability, as demonstrated by the first and last few cycles shown in the figure’s inset. The electrode retained 73% of its initial specific capacitance with a 96% coulombic efficiency after 3000 cycles. There was no apparent IR drop during the cycling process, and the charge-discharge patterns stayed almost symmetrical, demonstrating exceptional cycling stability. As shown in Fig. 10f, XRD patterns before and after electrochemical stability are highly consistent. The characteristics hkl planes of the active material at (220), (311), (222), (400), (422), (511), and (440) remains clearly visible and located at their original position without any significant shifting. Furthermore, the sharp high intensity peak (111) and (200) corresponds to the nickel foam, which also shows no sign off no phase transformation.

### Antifungal activity

**Fig. 11:**
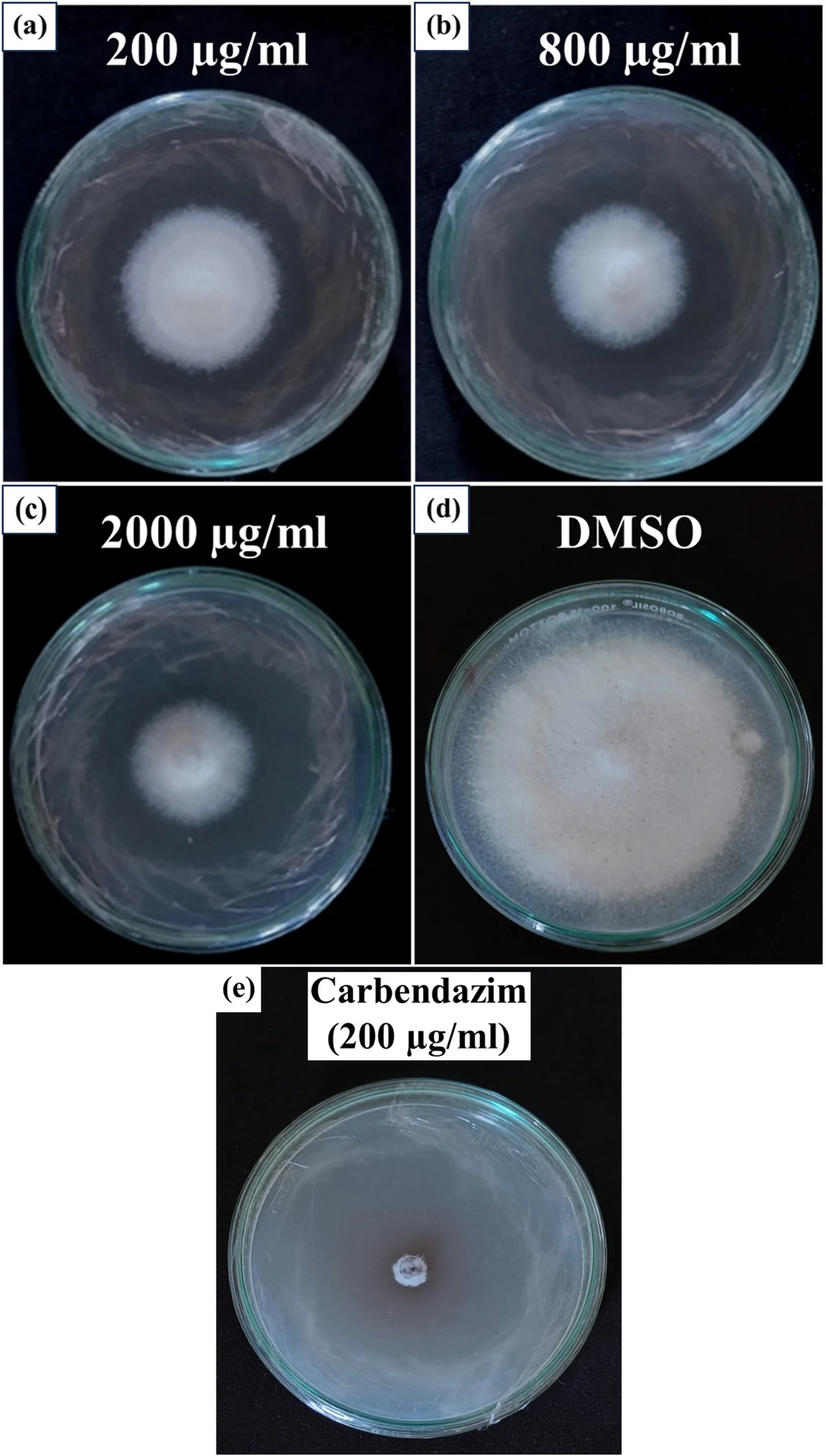
Evaluation of antifungal potential of the co-precipitation derived CoFe-3 against the fungi strain.

**Fig. 12:**
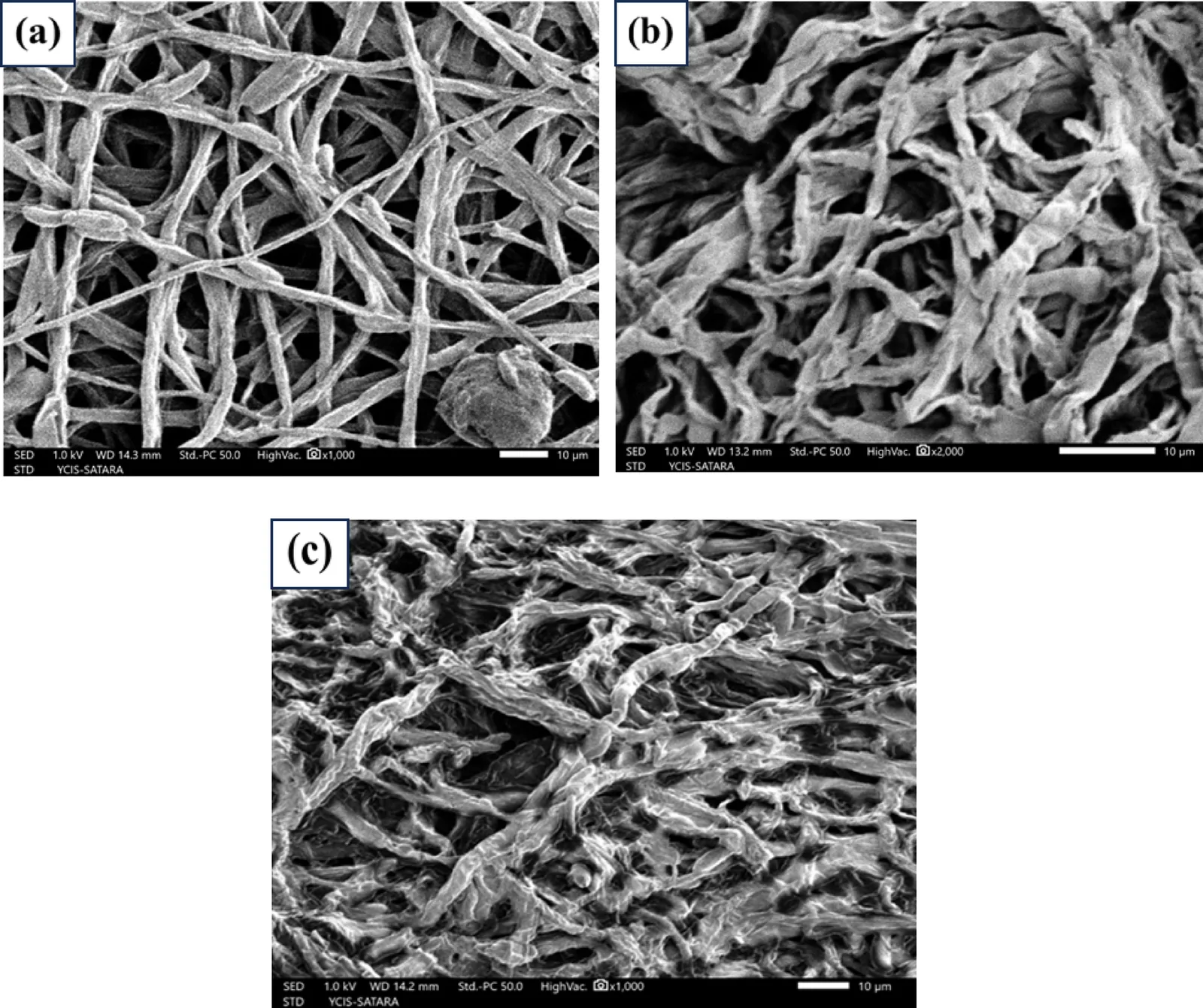
Comparative SEM images illustrating post-treatment morphological changes of *F. Solani* (a) DMSO, (b) CoFe-3 NCs, (c) Carbendazim.

*Fusarium solani (F. solani)* was chosen as a model fungus to study the antifungal properties of the CoFe-3, which heavily depends on their physiochemical properties. NCs with well-developed hexagonal shape and larger surface area strengthens the fungal cell attachment and promotes greater ROS production^82^. Improved crystallinity combined with the Fe and Co oxidation states improves redox performance and boosts metal-ion release, thereby intensifying the oxidative stress against fungi^83^. Collectively, these attributes leads to membrane disruption and yield markedly improved antifungal activity. Figure 11a-d and Table 2 illustrate the inhibitory effect of CoFe-3 at three discrete concentrations (200, 800, and 2000 µg/ml) on the *F. solani* after 10th days. The day wise inhibitory effect of the sample (Fig. S6) has been reported in the Table S2 to S10. A DMSO-treated group serves as a negative control, exhibiting no detectable suppression of the fungal growth. When treated with the lowest concentration (200 µg/ml), *F.* solani demonstrated 37.57% inhibition after 10 days. Further raising the concentration of CoFe-3 to 800 µg/ml resulted in a 42.75% inhibitory percentage. Lastly, at the maximum tested concentration (2000 µg/ml), nearly 52.41% inhibition of the *F. solani* was observed. To assure the experimental reliability, carbendazim was included as a standard positive control at 200 µg/ml. As depicted in Fig. 11e, the treatment resulted in a distinct zone of inhibition indicating the restricted fungal growth. However, an expanded zone of inhibition and radial growth was observed as the CoFe-3 concentration was increased. Hence, a dose-dependent effect of CoFe-3 NCs was observed in this finding. The overall result indicates that the prepared CoFe-3 NCs suppress *F. solani* and exhibit promising antifungal activity. Additionally, the SEM imaging was utilized to gain deeper insight into the antifungal efficacy of the CoFe-3 NCs. The morphological mycelial alteration of *F. solani* induced with DMSO and CoFe-3, fungicide treatment has been illustrated in Fig. 12. For DMSO treated *F. solani*, a well-developed conidiation and a uniform cylindrical mycelial surface with typical structural characteristics are evident as presented in Fig. 12a. In contrast, the exposure of *F. solani* to CoFe-3 NCs results in a loss of their smoothness and filamentous hyphal deformations and irregular mycelial shrinkages, as revealed in Fig. 12b^84^. Meanwhile, intensified mycelial shrinkages were observed in Fig. 12c after treatment with the fungicide.

**Table 2:** Inhibition (%) of radical growth of F. Solani after the treatment of CoFe-3.

Concentration (µg/ml)	Radial growth in mm (10th Day)				
	T1	T2	T3	Average	Inhibition (%)
200	62	59	55	58.66 ± 3.51	34.57
800	51	55	48	51.33 ± 3.51	42.75
2000	46	42	40	42.66 ± 3.05	52.41
Positive control (Carbendazim)	7.9	08	8.2	08.03 ± 0.15	91.04
Negative control (DMSO)	90	89	90	89.66 ± 0.57	0

Values are means of three replicate determination ± standard deviation.

Across the treatment, *F. solani* exhibits a diverse level of hyphal and structural damage, with the induced stress primarily affects the fungal cell wall. Fungal cell walls are well highly complex and intricate structure, mainly composed of polysaccharides, including chitin, glucans, mannans, and glycoproteins^84^. Among them, chitin serves as a key structural element of the fungal cell wall; thus, disruption of the chitin leads to malformed cells and osmotic imbalance. Accordingly, the captured SEM image (Fig. 12c) revealed mycelial deformation induced by CoFe-3, likely due to disrupted chitin biosynthesis^84^.

## Conclusion

The extensive investigation on the CoFe NCs exist, however; impact of their compositional variation on their multifunctional performance remains insufficiently explored. This study fills that gap by systematically correlating the structural features and its functional properties. A simple co-precipitation approach was applied to fabricate a series of Cobalt ferrite batches (CoFe-1, CoFe-2, CoFe-3, and CoFe-4). The influence of varying cobalt concentrations on physicochemical properties was systematically studied by various characterization techniques (XRD, BET, FESEM, TEM, and EDX). The functional performance of synthesized samples was evaluated across multiple applications, including dye degradation, energy storage, and antifungal activity. Among tested samples, the CoFe-3 batch was chosen for detailed study as it contains optimal cobalt concentration (0.1 M). CoFe-3 achieved 91.44% CV degradation in just 40 min under photo-Fenton optimal conditions. As a part of reliability, it maintained 81.73% efficiency after four reused cycles. Additionally, the CoFe-3 electrode showed a high specific capacitance of 614 F/g, demonstrating its exceptional charge storage capacity. Along with having a large capacitance, the electrode demonstrated remarkable electrochemical performance, delivering 15.04 Wh/kg of energy density at a corresponding power density of 217.16 W/kg. After 3000 continuous charge-discharge cycles at 5 A/g, the CoFe-3 electrode retained 73% of its initial capacitance and maintained a coulombic efficiency of 96%, demonstrating the material’s durability in long-term cyclic stability testing. Also, it successfully inhibits the radial growth of the fungi *F. Solani.* The SEM observations suggests pronounced morphological deformities in the fungal hyphae, indicating disruption of cell integrity induced by the CoFe. This research has been able to fill the research gap identified since it shows that the controlled compositional tuning of cobalt ferrite allows the simultaneous optimization of structural, electronic and surface properties resulting in high multifunctional performance. The results demonstrate a strong blueprint-property-function correlation, and the scientific and practical importance of CoFe-3 as a scaleable material in environmental remediation, energy storage and biomedical applications.

Despite the favorable outcomes, some constrains of this study needs to be addressed. The findings were conducted under lab-scale condition, which may differ in real wastewater scenario. Furthermore, the large-scale implementation needs to be explored.

## Supplementary Information

Below is the link to the electronic supplementary material.


Supplementary Material 1



Supplementary Material 2


## Data Availability

The data supporting the findings of this study are available in the supplementary material of this article, while additional data can be obtained from the corresponding author upon reasonable request.
