# Supplementary material for: Multifunctional cobalt ferrite nanoparticles with optimized cobalt doping for enhanced photo-Fenton catalysis, energy storage, and antifungal applications
Source: Sci Rep. 2026 Jun 14;16:27214. doi: 10.1038/s41598-026-55577-8 (PMC13526820; doi:10.1038/s41598-026-55577-8)
Supplement: Supplementary file 2 [file 41598_2026_55577_MOESM2_ESM.docx]

**Multifunctional Cobalt Ferrite Nanoparticles with Optimized Cobalt Doping for Enhanced Photo-Fenton Catalysis, Energy Storage, and Antifungal Applications**

Rupali Chavan^1^, Shruti Deshpande^2^, Jayram Shelke^3^, Vijay Chavan^4^, Mahendra Waghmare^3^, Prashant Patil^2^, Jyoti Jadhav^5^, Rahul Patil^6^, Deok-kee Kim^4*^ and Ashok Chougale^1*^

^1^Department of Chemistry, The New College Kolhapur, Shivaji University, Kolhapur, India.

^2^Department of Physics, The New College Kolhapur, Shivaji University, Kolhapur, India.

^3^Department of Botany, The New College Kolhapur, Shivaji University, Kolhapur, India.

^4^Semiconductor System Engineering Department and Convergence Engineering for Intelligent Drone, Sejong University, Seoul, Republic of Korea

^5^Department of Biochemistry, Shivaji University, Kolhapur, India.

^6^Department of Physics, Yashwantrao Patil Science College, Solankur, Shivaji University Kolhapur, India.

***Corresponding Authors**

Dr. Ashok D. Chougale

Email:[ashokdchougale@gmail.com](mailto:ashokdchougale@gmail.com), [ashokdchougale@newcollege.ac.in](mailto:ashokdchougale@newcollege.ac.in)

Prof. Deok-kee Kim

Email: [deokkeekim@sejong.ac.kr](mailto:deokkeekim@sejong.ac.kr)

**Materials**

Cobalt Chloride Hexahydrate (98%, CoCl_2_.6H_2_O), NaOH (98%) and D-(+)-Glucose (99.5%), and Hydrogen peroxide of 30% w/v were purchased from LOBA CHEMIE PVT. LTD. Ferric Chloride Hexahydrate (98%, FeCl_3_.6H_2_O) was bought from Sisco Research Laboratories Pvt. Ltd. All analytical purity reagents were received and utilized without any additional purification process.

**Synthesis of cobalt ferrite**

The facile co-precipitation method was used to prepare a designated CoFe batches (CoFe-1, CoFe-2, CoFe-3, and CoFe-4). The different stoichiometric ratios of two metal chloride solutions, FeCl_3_.6H_2_O (0.2 M) and CoCl_2_.6H_2_O (0.025 M, 0.05 M, 0.1 M, 0.15 M), were mixed with continuous stirring at 80°C for 30 minutes. After achieving homogeneous mixture, 0.2 M glucose solution was added in the reaction mixture of the metal chloride solution. Subsequently, the precipitation agent NaOH (0.1 M) was added dropwise to attain a pH-9 and incubated it for 2 hours with continuous stirring, thereby inducing nucleation and growth of CoFe precipitate. The resulting precipitates were collected by centrifugation, drying, and calcinating at 400°C for 2 hours. Finally, the obtained CoFe NCs were repeatedly washed with distilled water and alcohol, and used for further studies.

**Characterization**

The crystallinity and phase purity of the photocatalyst were performed with Cu-Kα radiation and using a 30 to 80 scanning 2θ degree X-ray diffraction (XRD), AXS D8 Advance) and λ ~1.54 A.U. The field emission scanning electron microscopy (FESEM), (Hitachi SU 8010 and JEOL JSM-7001F) were employed to investigate the surface morphological structure of the catalyst. The elemental composition was illustrated by performing the electron diffraction spectroscopy (EDS) technique. Further, Brunauer–Emmett–Teller (BET) (AutosorbiQ Station 2) was used to examine the average surface area a pore size distribution of the catalyst. To gain further insight into the microstructure, transmission electron microscopy (TEM) analysis was performed using a Hitachi/HF-3300 TEM at 300 kV.

**Dye degradation by photo-Fenton process**

The photocatalytic activity of a series of CoFe batches (CoFe-1, CoFe-2, CoFe-3, and CoFe-4) was tested with the photo-Fenton process for dye degradation. The 250 W visible light source was used to evaluate the photocatalytic activity of the catalyst. In a typical experiment, the photocatalytic glass reactor was used, containing 100 mg/L of the catalyst and 50 ml (0.1 mM) dye (CV) solution. The addition of the 10 mM H_2_O_2_ under a visible light source initiates the dye degradation reaction. After every 10 minutes, the 2 mL sample was collected and magnetically separated to examine the absorbance at 584 nm by UV-Visible spectrophotometer.

**The electrode preparation**:

MetrohmAutolab PGSTAT204 was used to analyze the supercapacitive behavior of the synthesized CoFe-3 in a three-electrode configuration using the electrochemical workstation. A platinum (Pt) wire served as the auxiliary electrode, and an Ag/AgCl electrode as a reference electrode. The working electrode was made by mixing the active material CoFe-3, PVDF, and acetylene black in an 8:1:1 ratio. The mixture was crushed into a paste with NMP (N-methyl-2-pyrrolidone) and applied to a 1×1 cm^2^ nickel foam, which was then dried overnight at 60 °C and used as the working electrode. The electrolyte used was 2 M KOH.

**Antifungal Activity**

The antifungal activity of the CoFe NCs was investigated by measuring the diameter of fungal colonies of the fungus *Fusarium soleni*. Initially, different concentrations of the CoFe NCs were prepared as 200 µg/mL, 800 µg/mL, and 2000 µg/mL, respectively. Then, under aseptic conditions, 20-25 mL of Potato Dextrose Agar (PDA) media along with 1 mL of prepared NCs solution were transferred to sterile petri plates and allowed to solidify. Further, a 6 mm diameter mycelial disc was taken from the pure fungus culture and inoculated at the center of the petri plates. The PDA plate without NCs was used as a negative control, while the PDA plate with fungicide carbendazim was used as a positive control. Lastly, the rate of inhibition of the mycelia disc growth was calculated by using the following (Eq.1),

$Rate of Inhibition \left( \% \right)= \frac{R-r}{R}\times100$	(1)

where ‘R’ is the mycelial radial growth of control fungus culture, and ‘r’ represents the mycelial radial growth of fungus culture treated with the different CoFe NCs concentrations.

| **Table S1:** Comparative performance of reported magnetic nanocomposites-based materials for environmental remediation of wastewater | | | | | |
| --- | --- | --- | --- | --- | --- |
| **Catalyst** | **Target Pollutant** | **Treatment Mechanism** | **Time Period** | **Performance Efficiency** | **Ref.** |
| NiFe_2_O_4_ | Rhodamine B (RhB) | photo-Fenton | 210 min | ~50.99 % | ^55^ |
| ZnO/NiFe_2_O_4_ | Methylene Blue (MB) | Photocatalysis | 210 min | ~56.19 % | ^55^ |
| Fe_3_O_4_-loaded TiO_2_ | Methylene Blue (MB) | Photocatalysis | 120 min | ~100 % | ^56^ |
| MnFe_2_O_4_/g-C_3_N_4_ | Methylene Blue (MB) | photo-Fenton | 240 min | ~80 % | ^57^ |
| ZnFe_2_O_3_ | Methyl Orange (MO) | Photocatalysis | 180 min | ~90 % | ^58^ |
| MgAC-Fe_3_O_4_ [0.2 g]/TiO_2_ | Methylene Blue (MB) | photo‑Fenton | 180 min | ~17 % | ^59^ |
| MgAC-Fe_3_O_4_ [0.1 g]/TiO_2_ | Methylene Blue (MB) | photo‑Fenton | 180 min | ~92 % | ^59^ |
| GO–ZnFe_2_O_4_ | Reactive Synozol Red | photo‑Fenton | 60 min | ~94–96 % | ^60^ |
| CuFe_2_O_4_ | Methylene Blue (MB) | photo‑Fenton | 60 min | ~91 % | ^61^ |
| ZnFe_2_O_4_ | Reactive Synozol Red | photo‑Fenton | 60 min | ~57 % | ^60^ |
| CuFe_2_O_4_/TA | Methylene Blue (MB) | photo‑Fenton | 80 min | 92.1 % | ^62^ |
| MgFe_2_O_4_ | Methylene Blue (MB) | photo‑Fenton | 30 min | ~95-98 % | ^63^ |
| CoFe_2_O_4_ | Crystal Violet (CV) | photo‑Fenton | 40 min | ~91.44 % | Current Study |

|  |  |
| --- | --- |
| **Fig. S1:** Elemental compositional analysis of CoFe-3 by EDX | |

**Fig. S2:** Zero-point pH of Cobalt ferrite nanocomposites

**Fig. S3:** Radical scavenging effect showing predominant role of reactive species (•O_2_^-^ and •OH)

|  |  |
| --- | --- |
| **Fig. S4:** Catalytic stability of CoFe-3 (a) effect of existing ions; and (b) practical water test | |

|  |  |
| --- | --- |
| **Fig. S5:** (a) CV curve of CoFe-3 electrode recorded in non -Faradic region at different scan rate; and (b) Plot of current density Vs. scan rate to estimate Cdl value | |

| **Daily Radial growth** | **Varied CoFe-3 concentration** | | | |
| --- | --- | --- | --- | --- |
|  | **200 µg/ml** | **800 µg/ml** | **2000 µg/ml** | **DMSO** |
| 1^st^ |  |  |  |  |
| 2^nd^ |  |  |  |  |
| 3^rd^ |  |  |  |  |
| 4^th^ |  |  |  |  |
| 5^th^ |  |  |  |  |
| 6^th^ |  |  |  |  |
| 7^th^ |  |  |  |  |
| 8^th^ |  |  |  |  |
| 9^th^ |  |  |  |  |
| **Fig. S6:** The antifungal activity of the Synthesized CoFe-3 against the fungi *F. Solani* | | | | |

| **Table S2:** Inhibition (%) of radical growth of *F. Solani* after the treatment of CoFe-3 | | | | | |
| --- | --- | --- | --- | --- | --- |
| **Concentration (µg/ml)** | **Radial growth in mm (1^st^ Day)** | | | | |
|  | **T1** | **T2** | **T3** | **Average** | **Inhibition (%)** |
| 200 | 00 | 00 | 00 | 00.00 ± 00.00 | 100 |
| 800 | 00 | 00 | 00 | 00.00 ± 00.00 | 100 |
| 2000 | 00 | 00 | 00 | 00.00 ± 00.00 | 100 |
| Positive Control (Carbendazim) | 00 | 00 | 00 | 00.00 ± 00.00 | 100 |
| Negative Control (DMSO) | 00 | 00 | 00 | 00.00 ± 00.00 | 100 |
| Values are means of three replicate determination ± standard deviation. | | | | | |

| **Table S3:** Inhibition (%) of radical growth of *F. Solani* after the treatment of CoFe-3 | | | | | |
| --- | --- | --- | --- | --- | --- |
| **Concentration (µg/ml)** | **Radial growth in mm (2^nd^ Day)** | | | | |
|  | **T1** | **T2** | **T3** | **Average** | **Inhibition (%)** |
| 200 | 10 | 10 | 08 | 09.33 ± 1.15 | 89.59 |
| 800 | 09 | 09 | 08 | 08.66 ± 0.57 | 90.33 |
| 2000 | 00 | 00 | 00 | 00.00 ± 00.00 | 00 |
| Positive Control (Carbendazim) | 00 | 00 | 00 | 00.00 ± 00.00 | 100 |
| Negative Control (DMSO) | 20 | 18 | 21 | 19.66 ± 1.52 | 00 |
| Values are means of three replicate determination ± standard deviation. | | | | | |

| **Table S4:** Inhibition (%) of radical growth of *F. Solani* after the treatment of CoFe-3 | | | | | |
| --- | --- | --- | --- | --- | --- |
| **Concentration (µg/ml)** | **Radial growth in mm (3^rd^ Day)** | | | | |
|  | **T1** | **T2** | **T3** | **Average** | **Inhibition (%)** |
| 200 | 17 | 18 | 17 | 17.33 ± 0.57 | 80.66 |
| 800 | 15 | 14 | 15 | 14.66 ± 0.57 | 83.98 |
| 2000 | 10 | 12 | 13 | 11.66 ± 1.52 | 86.98 |
| Positive Control (Carbendazim) | 00 | 00 | 00 | 00.00 ± 00.00 | 100 |
| Negative Control (DMSO) | 30 | 32 | 33 | 31.66 ± 1.52 | 64.68 |
| Values are means of three replicate determination ± standard deviation. | | | | | |

| **Table S5:** Inhibition (%) of radical growth of *F. Solani* after the treatment of CoFe-3 | | | | | |
| --- | --- | --- | --- | --- | --- |
| **Concentration (µg/ml)** | **Radial growth in mm (4^th^ Day)** | | | | |
|  | **T1** | **T2** | **T3** | **Average** | **Inhibition (%)** |
| 200 | 24 | 26 | 23 | 24.33 ± 1.52 | 72.86 |
| 800 | 21 | 24 | 17 | 20.66 ± 3.51 | 76.95 |
| 2000 | 16 | 17 | 14 | 15.66 ± 1.52 | 82.52 |
| Positive Control (Carbendazim) | 00 | 00 | 00 | 00.00 ± 00.00 | 100 |
| Negative Control (DMSO) | 41 | 43 | 38 | 40.66 ± 2.51 | 54.64 |
| Values are means of three replicate determination ± standard deviation. | | | | | |

| **Table S6:** Inhibition (%) of radical growth of *F. Solani* after the treatment of CoFe-3 | | | | | |
| --- | --- | --- | --- | --- | --- |
| **Concentration (µg/ml)** | **Radial growth in mm (5^th^ Day)** | | | | |
|  | **T1** | **T2** | **T3** | **Average** | **Inhibition (%)** |
| 200 | 28 | 30 | 32 | 30 ± 2 | 66.54 |
| 800 | 25 | 26 | 26 | 25.66 ± 0.57 | 71.37 |
| 2000 | 19 | 23 | 20 | 20.66 ± 2.08 | 76.95 |
| Positive Control (Carbendazim) | 00 | 00 | 00 | 00.00 ± 00.00 | 100 |
| Negative Control (DMSO) | 50 | 46 | 52 | 49.33 ± 3.05 | 44.98 |
| Values are means of three replicate determination ± standard deviation. | | | | | |

| **Table S7:** Inhibition (%) of radical growth of *F. Solani* after the treatment of CoFe-3 | | | | | |
| --- | --- | --- | --- | --- | --- |
| **Concentration (µg/ml)** | **Radial growth in mm (6^th^ Day)** | | | | |
|  | **T1** | **T2** | **T3** | **Average** | **Inhibition (%)** |
| 200 | 37 | 35 | 39 | 37 ± 2 | 58.73 |
| 800 | 33 | 29 | 32 | 31.33 ± 2.08 | 65.05 |
| 2000 | 27 | 23 | 24 | 24.66 ± 2.08 | 72.49 |
| Positive Control (Carbendazim) | 1.2 | 0.99 | 1.5 | 1.23 ± 0.25 | 98 |
| Negative Control (DMSO) | 60 | 63 | 59 | 60.66 ± 2.08 | 32.34 |
| Values are means of three replicate determination ± standard deviation. | | | | | |

| **Table S8:** Inhibition (%) of radical growth of *F. Solani* after the treatment of CoFe-3 | | | | | |
| --- | --- | --- | --- | --- | --- |
| **Concentration (µg/ml)** | **Radial growth in mm (7^th^ Day)** | | | | |
|  | **T1** | **T2** | **T3** | **Average** | **Inhibition (%)** |
| 200 | 42 | 45 | 40 | 42 ± 2 | 52.78 |
| 800 | 36 | 39 | 35 | 36.66 ± 0.57 | 59.10 |
| 2000 | 25 | 28 | 32 | 28.33 ± 2.08 | 68.40 |
| Positive Control (Carbendazim) | 3.5 | 3.2 | 3.8 | 3.5 ± 0.3 | 96.09 |
| Negative Control (DMSO) | 66 | 68 | 71 | 68.33 ± 3.05 | 23.79 |
| Values are means of three replicate determination ± standard deviation. | | | | | |

| **Table S9:** Inhibition (%) of radical growth of *F. Solani* after the treatment of CoFe-3 | | | | | |
| --- | --- | --- | --- | --- | --- |
| **Concentration (µg/ml)** | **Radial growth in mm (8^th^ Day)** | | | | |
|  | **T1** | **T2** | **T3** | **Average** | **Inhibition (%)** |
| 200 | 46 | 51 | 47 | 48 ± 2 | 46.46 |
| 800 | 40 | 43 | 39 | 40.66 ± 0.57 | 54.64 |
| 2000 | 35 | 30 | 33 | 32.66 ± 2.08 | 63.56 |
| Positive Control (Carbendazim) | 5 | 5.3 | 5.5 | 5.2 ± 0.25 | 94.12 |
| Negative Control (DMSO) | 74 | 78 | 71 | 74.33 ± 3.05 | 17.10 |
| Values are means of three replicate determination ± standard deviation. | | | | | |

| **Table S10:** Inhibition (%) of radical growth of *F. Solani* after the treatment of CoFe-3 | | | | | |
| --- | --- | --- | --- | --- | --- |
| **Concentration (µg/ml)** | **Radial growth in mm (9^th^ Day)** | | | | |
|  | **T1** | **T2** | **T3** | **Average** | **Inhibition (%)** |
| 200 | 53 | 56 | 51 | 53.33 ± 2 | 40.52 |
| 800 | 46 | 49 | 45 | 46.66 ± 0.57 | 47.95 |
| 2000 | 35 | 40 | 38 | 37.66 ± 2.08 | 57.99 |
| Positive Control (Carbendazim) | 7.9 | 7.3 | 7.8 | 7.6 ± 0.3 | 91.44 |
| Negative Control (DMSO) | 81 | 84 | 77 | 80.66 ± 3.05 | 10.03 |
| Values are means of three replicate determination ± standard deviation. | | | | | |
